# Lipid remodeling regulator 1 (LRL1) is differently involved in the phosphorus‐depletion response from PSR1 in *Chlamydomonas reinhardtii*

**DOI:** 10.1111/tpj.14473

**Published:** 2019-08-23

**Authors:** Nur A. Hidayati, Yui Yamada‐Oshima, Masako Iwai, Takashi Yamano, Masataka Kajikawa, Nozomu Sakurai, Kunihiro Suda, Kanami Sesoko, Koichi Hori, Takeshi Obayashi, Mie Shimojima, Hideya Fukuzawa, Hiroyuki Ohta

**Affiliations:** ^1^ Graduate School of Bioscience and Biotechnology Tokyo Institute of Technology 4259‐B‐65 Nagatsuta‐cho, Midori‐ku Yokohama 226‐8501 Japan; ^2^ School of Life Science and Technology Tokyo Institute of Technology 4259‐B‐65 Nagatsuta‐cho, Midori‐ku Yokohama 226‐8501 Japan; ^3^ Graduate School of Biostudies Kyoto University Kyoto 606‐8502 Japan; ^4^ Technology Development Kazusa DNA Research Institute Kazusa‐kamatari 2‐6‐7 Kisarazu Chiba 292‐0818 Japan; ^5^ Graduate School of Information Sciences Tohoku University 6‐3‐09, Aramaki‐Aza‐Aoba, Aoba‐ku Sendai 980‐8679 Japan; ^6^ Present address: National Institute of Genetics Bioinformation & DDBJ Center 1111 Yata Mishima Shizuoka 411‐8540 Japan

**Keywords:** *Chlamydomonas reinhardtii*, transcription factor, lipid remodeling, phosphorus starvation, co‐expression analysis

## Abstract

The elucidation of lipid metabolism in microalgae has attracted broad interest, as their storage lipid, triacylglycerol (TAG), can be readily converted into biofuel via transesterification. TAG accumulates in the form of oil droplets, especially when cells undergo nutrient deprivation, such as for nitrogen (N), phosphorus (P), or sulfur (S). TAG biosynthesis under N‐deprivation has been comprehensively studied in the model microalga *Chlamydomonas reinhardtii*, during which TAG accumulates dramatically. However, the resulting rapid breakdown of chlorophyll restricts overall oil yield productivity and causes cessation of cell growth. In contrast, P‐deprivation results in oil accumulation without disrupting chloroplast integrity. We used a reverse genetics approach based on co‐expression analysis to identify a transcription factor (TF) that is upregulated under P‐depleted conditions. Transcriptomic analysis revealed that the mutants showed repression of genes typically associated with lipid remodeling under P‐depleted conditions, such as sulfoquinovosyl diacylglycerol 2 (*SQD2*), diacylglycerol acyltransferase (*DGTT1*), and major lipid droplet protein (*MLDP*). As accumulation of sulfoquinovosyl diacylglycerol and TAG were suppressed in P‐depleted mutants, we designated the protein as lipid remodeling regulator 1 (LRL1). *LRL1* mutants showed slower growth under P‐depletion. Moreover, cell size in the mutant was significantly reduced, and TAG and starch accumulation per cell were decreased. Transcriptomic analysis also suggested the repression of several genes typically upregulated in adaptation to P‐depletion that are associated with the cell cycle and P and lipid metabolism. Thus, our analysis of LRL1 provides insights into P‐allocation and lipid remodeling under P‐depleted conditions in *C. reinhardtii*.

**Open Research Badges:**

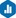

This article has earned an Open Data Badge for making publicly available the digitally‐shareable data necessary to reproduce the reported results. The sequencing data were made publicly available under the BioProject Accession number PRJDB6733 and an accession number LC488724 at the DNA Data Bank of Japan (DDBJ). The data is available at https://trace.ddbj.nig.ac.jp/BPSearch/bioproject?acc=PRJDB6733; http://getentry.ddbj.nig.ac.jp/getentry/na/LC488724. The metabolome data were made publicly available and can be accessed at http://metabolonote.kazusa.or.jp/SE195:/; http://webs2.kazusa.or.jp/data/nur/.

## Significance Statement

We used co‐expression analysis − an approach to predict the function of unknown genes based on biological processes − in *Chlamydomonas reinhardtii* to identify a putative transcription factor, lipid remodeling regulator 1 (LRL1), that is involved in the process of adapting to phosphorus‐deficient conditions by concomitantly regulating the cell cycle, phosphorus, and lipid metabolism.

## Introduction

Microalgae are a promising source of biofuel feedstock as they produce triacylglycerol (TAG) as a major storage lipid, especially under nutrient‐depleted conditions (Hu *et al*., 2008; Siaut *et al*., 2011). TAG is mainly constituted by three molecules of fatty acid that are esterified to one molecule of glycerol, thus it can be readily used as a biodiesel precursor to form fatty acid methyl ester (Merchant *et al*., 2012). Manipulation of lipid metabolism‐associated genes has been used as a basic approach to evaluate changes in the lipid biosynthesis pathway. In some cases, however, this does not lead to enhanced TAG accumulation. For example, overexpression of the diacylglycerol acyltransferases (*CrDGTT1−3*) does not improve the TAG content and composition in *C. reinhardtii* (La Russa *et al*., 2012). Moreover, overexpression of two *C. reinhardtii* glycerol‐3‐phosphate dehydrogenases does not improve the overall TAG yield (Bajhaiya *et al*., 2016). A metabolic approach that uses the promoter for sulfoquinovosyl diacylglycerol (*SQD2*), which is upregulated under P‐starvation, has successfully increased oil yield in *C. reinhardtii* under P‐deficient conditions (Iwai *et al*., 2014). The resulting 1.5‐ to 2‐fold increase in TAG in overexpression lines relative to control indicates the robustness of the regulatory control of downstream genes by the *SQD2* promoter. Phosphorus Starvation Response (PSR1), a well studied transcription factor (TF) that regulates P‐metabolism, has also proved to be a regulator for both TAG and starch accumulation in *C. reinhardtii*. PSR1 regulates some of the essential genes that are associated with lipid and starch biosynthesis (Bajhaiya *et al*., 2016). TAG accumulation is increased without affecting cell growth in the PSR1‐overexpressing strain CC‐125 (Ngan *et al*., 2015).

TAG accumulation is a complicated process in *C. reinhardtii*, and therefore additional TFs might be involved. The use of a low Pi−responsive promoter and a TF such as PSR1 for TAG overproduction has shed some light on the clarification of regulatory mechanisms involved in TAG synthesis under P‐starvation in *C. reinhardtii* (Ngan *et al*., 2015; Bajhaiya *et al*., 2016). Concomitantly with TAG accumulation, membrane remodeling is a typical response under P‐starvation and occurs widely throughout oxygenic photosynthetic organisms from cyanobacteria and phytoplankton to land plants (Nakamura *et al*., 2009; Hori *et al*., 2016; Shemi *et al*., 2016). In this response, phospholipids are replaced by non‐P glycolipids and/or betaine lipids, which releases inorganic phosphate from membranes, thus providing phosphate to other important cellular processes (Shimojima *et al*., 2013; Hori *et al*., 2016). Moreover, a regulatory gene involved in the transition from N‐depleted to N‐replete conditions, *Compromised Hydrolysis of Triacylglycerols 7* (*CHT7*), which may be a repressor of cellular quiescence, provides a mechanistic insight into how cells exit quiescence after nutrient deprivation (Tsai *et al*., 2014). To clarify the transcriptional regulation involved in TAG synthesis and membrane remodeling during P‐depletion, which may overcome the inverse correlation between biomass productivity and TAG accumulation in microalgae, targeting related TFs by transcriptomic profiling is essential, as they might induce or repress multiple genes in this pathway.

Co‐expression analysis has been useful for predicting regulatory relationships between previously unidentified TFs and genes that are involved in a particular metabolic pathway in model organisms (Aoki *et al*., 2016). In this study, a set of public transcriptomic data was used to identify candidate TFs that are co‐expressed with *CrDGTT1* under P‐depleted conditions (Aoki *et al*., 2016). Mutant analysis of a homolog of AtMYB64, an R2R3‐MYB TF, in *C. reinhardtii* that was characterized in this study, indicated an overall change in cell metabolism that affected not only TAG biosynthesis but also membrane remodeling under P‐starvation. This putative TF was highly upregulated during the later stage of P‐starvation. A transactivation assay in tobacco leaves showed a positive activation of *sulfoquinovosyl diacylglycerol 2* (*CrSQD2‐2*) when it was co‐expressed with a *transparent testa glabra 1* homolog (*CrTTG1*) and a basic helix−loop−helix homolog (*CrbHLH*) in *C. reinhardtii*. Therefore, we designated the AtMYB64 homolog as lipid remodeling regulator 1 (LRL1), as we conclude that this TF may act as a regulator for both membrane remodeling and TAG synthesis under P‐starvation.

## Results

### Identification of a putative TF by co‐expression analysis

In this study, we relied upon a co‐expression analysis to identify putative TFs in *C. reinhardtii* that are correlated with lipid metabolism under P‐depleted conditions. We selected *CrDGTT1* as the query gene for the co‐expression analysis, as it is involved in the *de novo* synthesis of TAG (Boyle *et al*., 2012; Li‐Beisson *et al*., 2015). Artificial miRNA knock‐down of *CrDGTT1* in *C. reinhardtii* suppresses TAG accumulation, thus affecting fatty acid composition and, in particular, polyunsaturated fatty acid composition under N‐starvation (Liu *et al*., 2016). In addition, under nutrient starvation such as N‐, P‐, or S‐starvation, *CrDGTT1* transcripts are increased, which reflects the stimulation of enhanced TAG biosynthesis (Miller *et al*., 2010; Boyle *et al*., 2012; Iwai *et al*., 2014; Sato *et al*., 2014). Regulatory genes that are co‐expressed when *CrDGTT1* is highly induced might regulate TAG accumulation.

**Figure 1:**
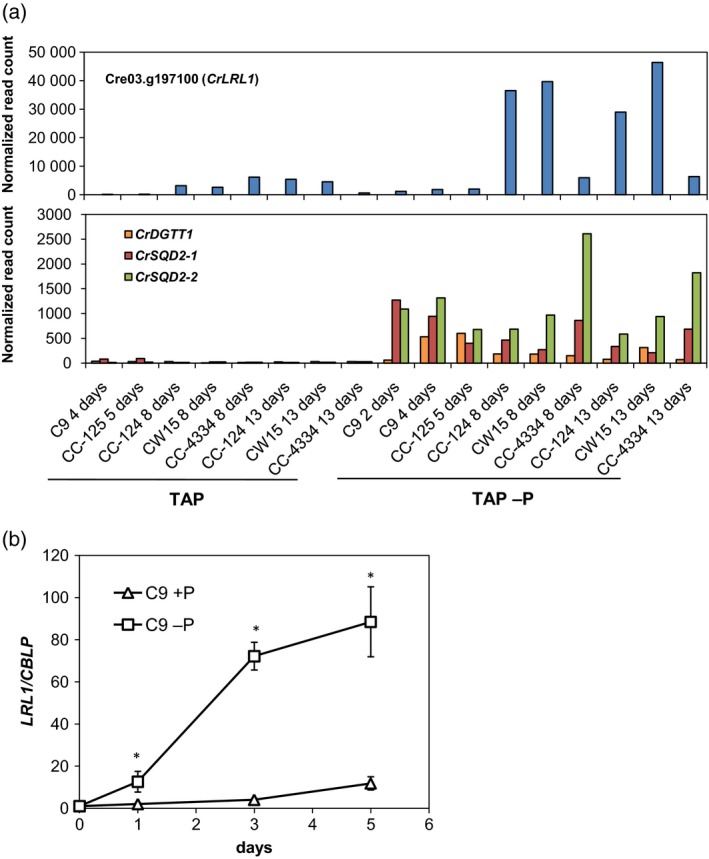
Identification of the gene Cre03.g197100, subsequently named Lipid Remodeling reguLator 1 (*LRL1*). (a) RNA‐seq data for Cre03.g197100 (*LRL1*) and genes involved in sulfolipid and TAG biosynthesis in various *Chlamydomonas reinhardtii* wild‐type (WT) strains (C9, CC‐125, CC‐124, CW15, CC‐4334) under P‐sufficient and P‐deficient conditions. TAP, Tris−acetate−phosphate medium; TAP −P, TAP without P. (b) Validation of *CrLRL1* expression under P‐sufficient and P‐deficient conditions by real‐time qPCR. Expression was normalized to that of *CBLP*. As shown by co‐expression analysis, *LRL1* expression increased in response to P‐depleted conditions during the later stage of P‐starvation as compared with P‐replete conditions, which leads to the hypothesis that upregulated expression of this gene is concomitant with the upregulation of *CrDGTT1* under P‐depletion. Data are shown as the mean ± SD from three biological replicates. Statistical significance was determined with the two‐tailed Student's *t*‐test; *P <* *0.05 relative to the corresponding WT cells.

Co‐expression analysis was performed as described in ALCOdb, an open co‐expression database based on previously published transcriptomics data for microalgae (Aoki *et al*., 2016). Genes that encode selected putative TF(s) that are co‐expressed with *CrDGTT1* were ranked based on Pearson's correlation coefficient and mutual rank (MR) with a public transcriptomic dataset (Table 1, Data S1). The highly co‐expressed putative TF genes were selected and then their expression under P‐depleted and P‐replete conditions was compared with RNA‐sequencing (RNA‐seq) analysis (see Table S1). A homolog of *Arabidopsis thaliana MYB64*, Cre03.g197100 (*LRL1*), showed the highest fold change under P‐depletion relative to P‐sufficient conditions (Table 1, Figure 1a). AtMYB64 and AtMYB119 act redundantly to regulate the FG5 transition and thus are involved in differentiation and cellularization during female gametogenesis in *A. thaliana* (Rabiger and Drews, 2013).

**Table 1:** Top‐ranked of putative transcription factors (TFs) from a co‐expression analysis of the gene encoding CrDGTT1 using a public transcriptomic dataset. Transcript level for the candidate TFs under P‐sufficient and P‐deficient conditions were obtained from RNA‐seq data described in Table S1

Gene ID	MR^a^	PCC^b^	*Arabidopsis thaliana* ortholog	*A. thaliana* annotation^c^	Transcript level +P (normalized read count)	Transcript level −P (normalized read count)	Fold change (−P/+P)
Cre02.g095750	6.63	0.8082	At5g67030	Zeaxanthin epoxidase (ZEP) (ABA1)	1519.557	4643.893	3.055
Cre12.g501450	46.5	0.7531	At3g55530	RING/U‐box superfamily protein	93.385	197.385	2.102
Cre01.g034350	46.96	0.7531	At5g02320	myb domain protein 3r‐5	37.354	144.483	3.793
Cre16.g673250	165.52	0.6651	At1g20980	Squamosa promoter binding protein‐like 14	933.848	811.77	0.869
Cre09.g417400	221.41	0.6827	At4g12620	Origin of replication complex 1B	187.517	220.947	1.177
Cre14.g612350	388.64	0.5386	At4g12620	Origin of replication complex 1B	132.98	316.528	2.37
Cre16.g654550	412.29	0.5252	At3g48430	Relative of early flowering 6	536.402	665.509	1.24
Cre16.g679050	423.77	0.5584	At4g12620	Origin of replication complex 1B	129.245	321.418	2.475
Cre03.g149350	445.47	0.4999	At5g66990	RWP‐RK domain‐containing protein	292.855	76.909	0.265
Cre14.g612100	475.93	0.5631	At5g66990	RWP‐RK domain‐containing protein	238.318	207.61	0.872
Cre03.g197100	615.54	0.5633	At5g11050	myb domain protein 64	192.746	2016.088	10.411
Cre07.g345350	660.42	0.5182	At5g67300	myb domain protein r1	158.381	114.252	0.723
Cre03.g182700	675.7	0.4666	At5g15850	CONSTANS‐like 1	38.101	41.789	1.094
Cre14.g620500	711.26	0.5146	At1g12980	Integrase‐type DNA‐binding superfamily protein	1435.885	2218.364	1.545

The normalized read count for candidate TF was compared for wild‐type CC‐125 cultures after 5 days under P‐sufficient (+P) and P‐deficient (−P) conditions. Fold change >1 indicates upregulation under P‐deficiency and fold change <1 indicates downregulation under P‐deficiency.

a Mutual rank.

b Pearson's correlation coefficient

c The short description of *A. thaliana* gene annotation based on the TAIR website.

To support the functional involvement of this putative TF, we carried out an investigation of LRL1 *in silico*. ChlamyNET, a *C. reinhardtii* gene co‐expression network (Romero‐Campero *et al*., 2016), identified LRL1 as being involved in core metabolic regulation and light response, which includes carbohydrate metabolic process, fatty acid biosynthesis process, and nitrogen compound metabolic process. Therefore LRL1 is considered as a highly authoritative hub and is co‐expressed with 87 other genes in this study, which underscores its regulatory importance (Romero‐Campero *et al*., 2016). Moreover, the Plant Transcription Factor Database (PlnTFDB) classified LRL1 as a MYB family protein, as it harbors a Myb DNA‐binding domain (Jin *et al*., 2017). Gene Ontology (GO) predicted its biological function to be possibly involved in cell cycle regulation and indicated that it is expressed under nutrient starvation. As expression of *CrDGTT1* hardly increases during the early stage of P‐depletion (Boyle *et al*., 2012), we analyzed the transcriptomics data under prolonged P‐depletion, during which *CrDGTT1* expression is induced and leads to TAG accumulation (Iwai *et al*., 2014). The expression profiler in ALCOdb provided the functionalities of the expression pattern of *CrDGTT1* and *LRL1*. The scatter plots clearly indicated that *CrDGTT1* and *LRL1* had a similar expression pattern, as both genes were induced and highly upregulated during the later stage of nutrient starvation (Figure S1). It is thus reasonable to choose this putative TF as a candidate TAG biosynthesis regulator and to further confirm the co‐expression result experimentally.

A time course analysis based on real‐time quantitative polymerase chain reaction (PCR) confirmed the RNA‐seq data (Figure 1b). Hierarchical clustering of the TF‐encoding genes from the 20 RNA‐seq samples listed in Figure S1 showed the separation of *LRL1* with other TF‐encoding genes in *C. reinhardtii* (Figure S6). LRL1 belongs to the TFs inducible by late‐phase P‐starvation. Meanwhile, PSR1 belongs to the TFs inducible by early phase P‐starvation. This segregation may be related to the different functions between PSR1 and LRL1 in regulating P‐starvation in *C. reinhardtii*.

For all relevant experiments, cells in logarithmic phase were initially transferred from P‐replete medium to promote the accumulation of TAG under P‐depleted conditions (Iwai *et al*., 2014). During P‐starvation, LRL1 expression was gradually increased as compared with its expression under P‐replete conditions, with a more significant increase observed among cells from early log phase (Figure 1b). The gradual response to P‐starvation might be correlated with the expression of genes that are also upregulated during early P‐starvation, such as *SQD2* (Figure 1a), whereas *SQD2* transcription is not upregulated after N‐deprivation (Boyle *et al*., 2012).

### Phylogenetic analysis of MYB proteins

**Figure 2:**
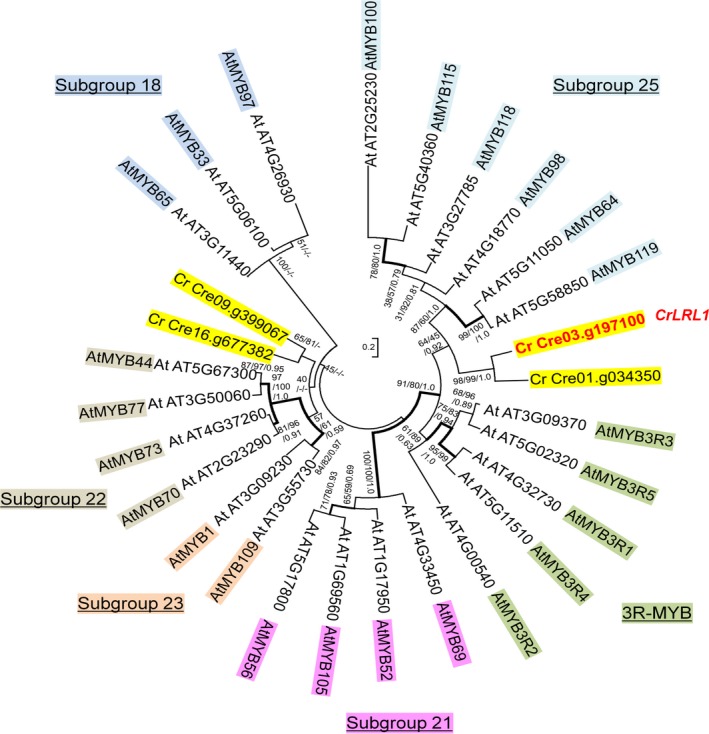
Phylogenetic analysis of MYB proteins. Some MYB proteins of *Arabidopsis thaliana* and *Chlamydomonas reinhardtii* were phylogenetically analyzed using maximum likelihood, neighbor‐joining, and Bayesian inference algorithms. The topologies and branch lengths were calculated using the maximum likelihood method based on the LG model +G (eight categories). Bootstrap values (maximum likelihood and neighbor‐joining) and Bayesian posterior probabilities (Bayesian inference) are indicated under each branch (maximum likelihood/neighbor‐joining/Bayesian inference). Branches with thick lines were highly supported by bootstrap values >70 and posterior probabilities >0.9. The symbol (−) indicates distinct topological arrangements. The scale bar represents 0.2 amino acid substitutions per site. The subgroups refer to those reported previously (Stracke *et al*., 2001).

Most MYB genes in eudicots, including those in *A. thaliana* and *Glycine max*, are classified as 2R‐MYB genes, whereas 3R‐MYB genes are clustered within one clade (Stracke *et al*., 2001). LRL1 retains partial W (tryptophan) repeats in the conserved N‐terminal DNA‐binding domain (the MYB domain), which is similar to its homolog AtMYB64 and is thus classified as an R2R3‐MYB gene (i.e., a2R‐MYB gene) (Figure 2). AtMYB64 retains its R2R3‐MYB repeats on the conserved N‐terminal DNA‐binding domain. Motif search with Pfam showed that the conserved motif detected was the MYB‐like DNA‐binding domain and included LRL1 in subgroup 25 of R2R3‐MYB genes, together with the AtMYB119 homolog (Figure 2). Based on protein homology searches using BLASTP from NCBI, *LRL1* had the highest level of sequence identity with *AtMYB64* (53.91% identity), whereas *AtMYB119* had 47.92% identity with *AtMYB64*. Thus, we determined that *LRL1* is the homolog of *AtMYB64*. Other MYB homologs in *C. reinhardtii* were identified in different subgroup with LRL1. Motif identification on Pfam showed that Cre09.g399067 and Cre16.g677382 were harbored the same R2R3‐MYB DNA‐binding domain as LRL1. Based on the GO, both are predicted to be involved in the abiotic stress responses, such as salt stress, ethylene, and abscisic acid. The conserved MYB‐like DNA‐binding domain is also featured across other land plants and microalgae species (Figure S2). The evolutionary conservation of the MYB binding domain suggested that we may be able to elucidate the function of this protein in various species.

### Characterization of LRL1 mutant strains

**Figure 3:**
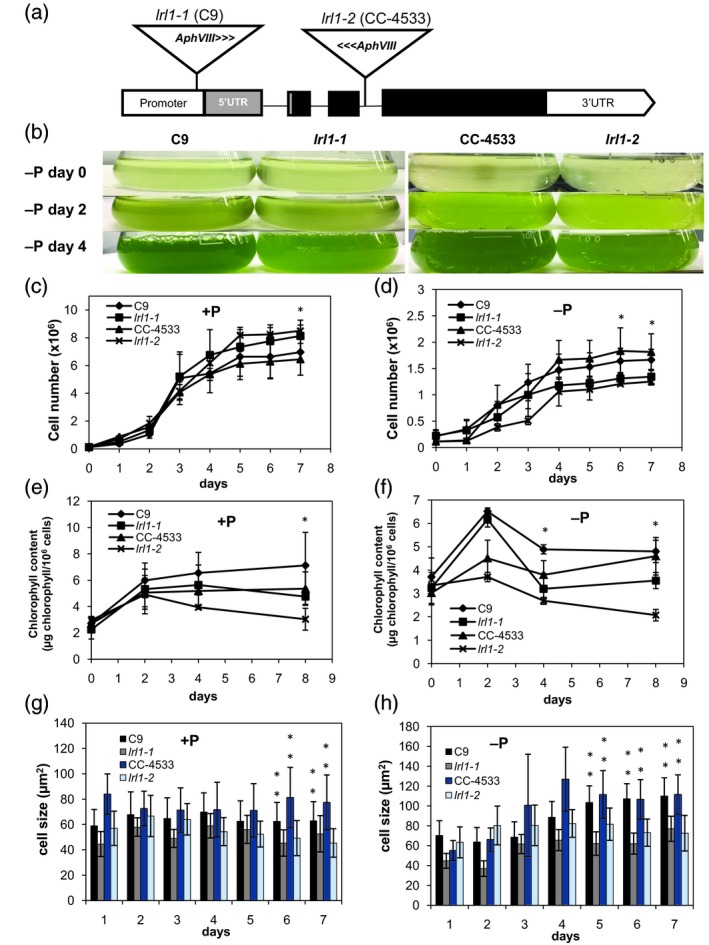
Analysis of the growth and cell size of *lrl1* mutants under P‐sufficient and P‐deficient conditions. (a) Schematic AphVIII‐tag positions in the Cre03.g197100 locus. (b) Comparison of control (C9 and CC‐4533) and *lrl1* mutants growing in liquid TAP −P medium (i.e., under P‐deficient conditions) with continuous light. (c, d) Growth curve of WT and *lrl1* mutants under (c) P‐sufficient and (d) P‐deficient conditions. Differences in culture coloration occurred after 4 days of growth under P‐starvation. (e, f) Chlorophyll content of WT and *lrl1* mutants under (e) P‐sufficient and (f) P‐deficient conditions. Data points indicate the mean value ± SD from at least three biological replicates. (g, h) Cell size of WT and *lrl1* mutants under (g) P‐sufficient and (h) P‐deficient conditions. Each culture was grown in three independent flasks. Cell size was measured from at least 100 single cells from all three independent flasks. Statistical significance was determined with the two‐tailed Student's *t*‐test; *P <* *0.05 and **P <* *0.01 relative to the corresponding WT cells.

We used two *Chlamydomonas* mutant libraries to obtain two mutants, *lrl1‐1* and *lrl1‐2*, both of which contained a disruption in *LRL1* (Figure 3a; Figure S3). Both mutants have two different control strains, which differ in their cell wall properties, C9 (CC‐408, wild‐type mt−) and CC‐4533 (cw15 mt−). We then examined phenotypic correlations between wild‐type (WT) and the *lrl1* mutants. For cells in logarithmic phase under P‐replete conditions, the resulting cell numbers for *lrl1‐1* and *lrl1‐2* were slightly higher relative to their respective parental lines (Figure 3c), but there was a significant decrease in their cell size (Figure 3g). Growth of *lrl1‐1* and *lrl1‐2* under P‐deficient conditions was slower relative to their respective parental lines (Figure 3d). The size of WT *C. reinhardtii* cells gradually increases during P‐starvation (Bajhaiya *et al*., 2016); however, this increase in the two mutants was markedly suppressed (Figure 3h). Moreover, chlorophyll content began to decrease after 2 days of P‐depletion, and the same trend was observed under P‐replete conditions, particularly when cells entered late logarithmic phase (Figure 3f). Decreased chlorophyll content in *lrl1‐1* and *lrl1‐2* cells may be associated with slower growth during the later stage of P‐starvation (Figure 3b).

### The *lrl1‐1* mutation affects several genes involved in lipid remodeling under P‐starvation

**Figure 4:**
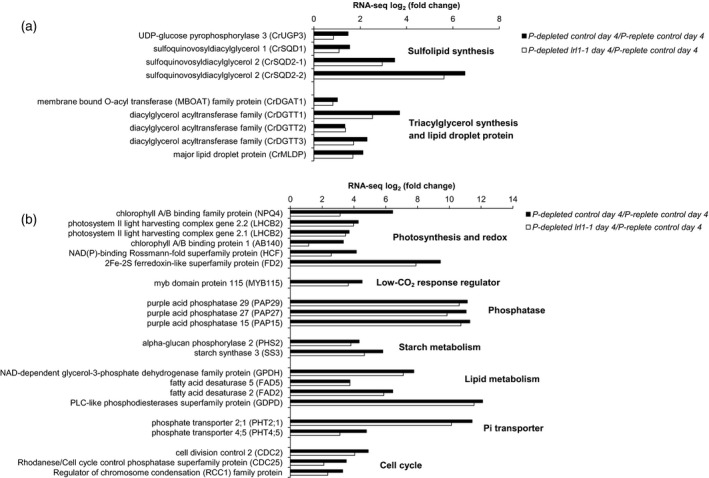
RNA‐seq analysis of WT and *lrl1‐1* after 4 days of P‐starvation. (a) Major genes involved in sulfolipid and TAG biosynthesis. (b) Differentially expressed genes (DEGs) that represent major genes involved in P‐starvation, as chosen from Data S2. Most of these genes showed upregulation during P‐starvation when compared with the P‐sufficient condition. Some genes in *lrl1‐1* were partially downregulated as compared with WT after 4 days of P‐starvation. Each gene was categorized based on specific annotation in GO or KOG terms.

To understand the phenotype noted above, we used RNA‐seq analysis to find out how repression of *LRL1* affects gene expression under P‐depleted conditions relative to P‐replete conditions. Analysis of the principal component analysis (PCA) clearly showed the separation of genes that were differentially expressed under P‐replete and P‐depleted conditions in PC2, whereas PC1 showed the separation of genes that were expressed during the early and late stages of P‐replete and P‐depleted conditions (Figure S7). Furthermore, we noted differences in the fold change of the expression of genes that correspond to sulfolipid biosynthesis, TAG biosynthesis, and lipid droplet protein (Figure 4a, Data S2 and S3).

TAG accumulation is one of the major events that occur when cells are deprived of P. Upon deficiency of a macronutrient such as N, P, or S, membrane glycerolipids are dramatically reduced, and, in contrast, TAG accumulates in lipid droplets (Boyle *et al*., 2012). In the case of genes related to TAG biosynthesis, all genes except for *CrDGTT4* were upregulated after 2 days of P‐starvation in WT, as compared with the P‐replete condition. In contrast, under the same culture conditions, most TAG biosynthesis‐related genes in *lrl1‐1* began to be repressed (Figures 4a and S9). After 4 days of P‐starvation, expression of *CrDGTT1* decreased by 20% in *lrl1‐1* relative to WT levels (Figure 4a). Thus, real‐time PCR was conducted to confirm the RNA‐seq result. As shown in Figure S9, significant repression of *CrDGTT1* occurred after 4 days of P‐starvation.

The gene for major lipid droplet protein (MLDP), a major structural protein for lipid droplet formation in *C. reinhardtii* (Moellering and Benning, 2010) showed a notable difference between WT and *lrl1‐1* cells under P‐deficient conditions as compared with P‐replete conditions (Figure S9). After 2 days of P‐starvation, *MLDP* expression in *lrl1‐1* was slightly lower relative to its expression in P‐replete cultures, whereas expression in WT increased up to three‐fold. Although expression of *MLDP* in *lrl1‐1* was gradually increased after 4 days of P‐starvation, the increase was not as high as that of WT (Figure S9). Real‐time PCR showed that suppression of MLDP in *lrl1‐1* occurred from 1 day after P‐starvation (Figure S9).

During the RNA‐seq analysis, another change was observed in genes involved in sulfolipid synthesis. Upregulation of sulfoquinovosyl diacylglycerol (SQDG) biosynthesis genes is a major event in the adaptation response to P‐starvation in photosynthetic organisms (Shimojima, 2011; Hori *et al*., 2016) and generally occurs under low P conditions to substitute for phospholipid breakdown (Essigmann *et al*., 1998; Hartel *et al*., 2000). *CrSQD1* and *CrSQD2* were repressed in *lrl1‐1* under P‐depletion (Figure 4). There are two copies of *SQD2* in *C. reinhardtii*, designated *CrSQD2‐1* and *CrSQD2‐2*, both of which are upregulated under P‐deficient conditions (Iwai *et al*., 2014; Bajhaiya *et al*., 2016). After 2 days of P‐starvation, Cr*SQD2‐1* and Cr*SQD2‐2* showed increased expression in WT and *lrl1‐1*, although expression in *lrl1‐1* was lower than in WT (Figure S9). After 4 days of P‐starvation, the decline in *CrSQD2‐1* and *CrSQD2‐2* expression was still observed (Figure 4; Figure S9). Real‐time PCR indicated that the changes in *CrSQD2‐1* and *CrSQD2‐2* expression initially began after 1 day of P‐starvation, with a significant difference observed after 4 days (Figure S9).

Overall, these results indicated that the major genes related to TAG biosynthesis were suppressed in *lrl1‐1* and *lrl1‐2*, and they also showed impairment of another lipid remodeling process, SQDG synthesis, in which phosphatidylglycerol (PG) is replaced by SQDG in the chloroplast membrane.

### Lipid and starch accumulation is repressed in *lrl1‐1* during P‐depleted conditions

**Figure 5:**
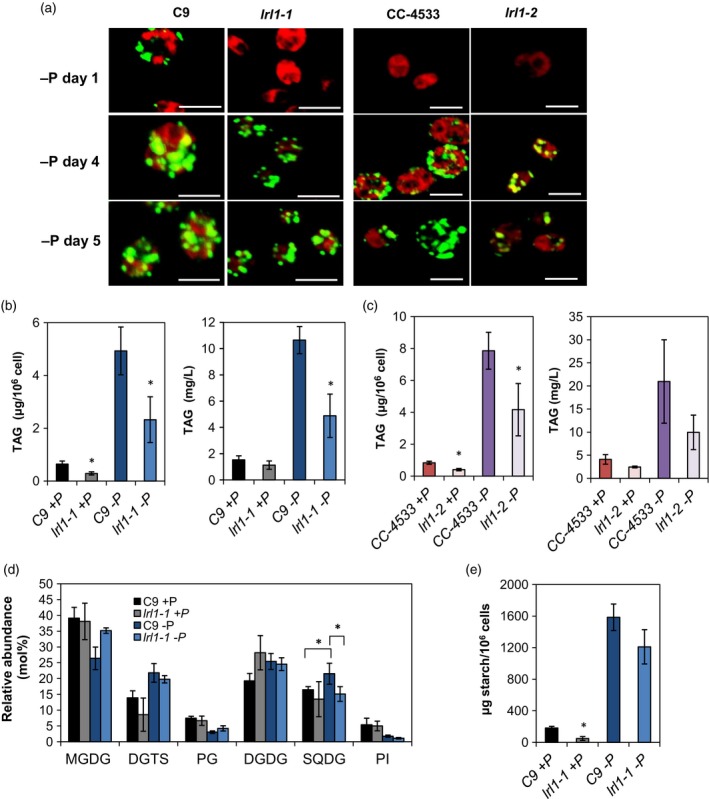
Lipid droplet, lipid, and starch analysis of WT and *lrl1* mutants. (a) Nile red staining at the designated days after cells were transferred to P‐depleted medium. Green fluorescence in the presence of Nile red indicates a neutral lipid, whereas red fluorescence indicates chlorophyll autofluorescence. Bars = 10 μm. (b,c) Triacylglycerol (TAG) quantification for (b) *lrl1‐1* and (c) *lrl1‐2* after 8 days in culture under P‐replete and P‐depleted conditions. TAG fatty acids were trans‐esterified by methanolysis, and then fatty acid moieties were measured by GC‐FID. (d) Quantification of membrane lipids from WT and *lrl1‐1* cells with two‐dimensional thin‐layer chromatography (TLC). Total fatty acids derived from each species were measured by GC‐FID. Abbreviations: monogalactosyl diacylglycerol (MGDG), digalactosyl diacylglycerol (DGDG), diacylglycerol‐*N*,*N*,*N*‐trimethylhomoserine (DGTS), sulfoquinovosyl diacylglycerol (SQDG), phosphatidylethanolamine (PE), phosphatidylserine (PS), phosphatidylinositol (PI), and phosphatidylglycerol (PG). (e) Starch quantification of WT and the *lrl1‐1* mutant after 8 days in culture. Data are shown as the mean ± SD from three biological replicates. Statistical significance was determined with the two‐tailed Student's *t*‐test; *P <* *0.05 relative to the corresponding WT cells as indicated by square brackets.

As a consequence of the impairment of genes associated with lipid remodeling in *lrl1* (Figures 4a and S9, and Data S2), the mole percent of SQDG decreased in the *lrl1* mutant, whereas no other notable changes were observed in other lipids except for slight increases in monogalactosyl diacylglycerol (MGDG) and PG, two other chloroplast lipids, in *lrl1‐1* as compared with WT under P‐depleted conditions (Figure 5d). There was no substantial change in lipid composition under P‐replete conditions (Figure 5d). RNA‐seq analysis also suggested that some fatty acid desaturase (FAD) genes might show different levels of expression in response to P‐depletion (Figure 4 and Data S3). The differences in *FAD2* transcripts might result in a change in the fatty acid composition of lipids, in which 18:2 fatty acids are more dominant in the major extraplastidic lipid diacylglycerol‐*N*,*N*,*N*‐trimethylhomoserine (DGTS) and less dominant in the major plastidic lipid digalactosyl diacylglycerol (DGDG) under P‐depleted conditions (Figure S5).

Because we screened primarily for TFs that are co‐expressed with *CrDGTT1*, the major TAG synthase in *C. reinhardtii*, we also looked for differences in TAG accumulation. The relationship between TAG accumulation and the defect in *LRL1* was initially observed through confocal microscopy by Nile red staining (Figure 5a). Because TAG accumulates to a greater extent under P‐deficient conditions, we used P‐deficient medium for the following experiments. Differences in lipid droplet accumulation occurred from 1 day after P‐starvation in *lrl1‐1*, although this phenotype was not observed in the WT line CC‐4533 (Figure 5a). The differences in lipid droplet formation between WT and *lrl1‐1* cells increased after 4 and 5 days of P‐starvation, with WT cells showing a greater cell size as compared with *lrl1‐1*. C9 WT cells were more likely to have an early response to P‐status relative to *lrl1‐1* cells. This delayed response was also observed in *lrl1‐2* relative to the CC‐4533 cells. TAG quantitative analysis by gas chromatography with a flame ionization detector (GC‐FID) showed that TAG content per cell was significantly decreased in *lrl1* mutants relative to WT under P‐deficient conditions (Figure 5b,c). Starch quantification showed a slight difference under P‐depleted conditions (Figure 5e), in agreement with a slight decrease in the expression of a starch synthase gene (*SS3*) in *lrl1‐1* (Figure 4b). The downregulation of *CrDGTT1* and *MLDP* expression in *lrl1‐1* (Figures 4a and S9) and *lrl1‐2* (Figure S9) may have resulted in impaired TAG synthesis under P‐depleted conditions, as the amount of TAG that accumulates in *C. reinhardtii* is associated with the abundance of MLDP (Tsai *et al*., 2014). However, there was no notable change in terms of the fatty acid profile associated with TAG (Figure S4). *De novo* fatty acid synthesis is maintained under P‐depleted conditions, as the major fatty acids associated with TAG under P‐depleted conditions are 16:0 and 18:1(9) as compared with P‐replete conditions. The inability of the *lrl1* mutants to increase in cell size may be associated with the decrease in the number of oil droplets and starch granules formed per cell under P‐depletion.

### Several genes involved in the P‐starvation response were repressed in *lrl1‐1*

Systematic overview of misregulated genes in *lrl1‐1* was done by comparing differential gene expression in control and *lrl1‐1* under P‐replete and P‐depleted conditions (Figure S8a,b,c). Out of 317 genes that were affected under P‐starvation, 150 were expressed at lower levels in *lrl1‐1* than in the control, only four of which had lower expression at day 2 (Figure S8d). This indicates that *lrl1‐1* might regulate the P‐starvation response genes during the later stage of starvation, whereas it has subtle regulational control during the earlier stage. Transcriptomics analysis showed that most of the genes that were involved in P‐homeostasis, starch and lipid metabolism, carbon concentrating mechanism, cell cycle, photosynthesis, and redox/electron transport were upregulated after 2 days of P‐starvation (Data S3). To distinguish the misregulated genes in the *lrl1‐1* mutant, MA plots were generated and indicated that several genes that were initially involved in the response to P‐starvation were repressed in the *lrl1‐1* mutant. Detailed RNA‐seq analysis in our study indicated that several genes that were regulated in a PSR1‐dependent manner also were repressed in the *lrl1‐1* mutant. Many of the genes encoding known phosphate transporters and purple acid phosphatases were repressed in *lrl1‐1* (Figure 4b). *GPDH* transcript was also repressed in *lrl1‐1*, indicating there may be a downregulation of the processes related to lipid and carbohydrate metabolism, which thus might affect the carbon supply for TAG and starch biosynthesis. A subset of genes involved in photosynthesis and redox/electron transport was also repressed in *lrl1‐1*, such as LHCB2, chlorophyll A/B binding protein, high‐chlorophyll fluorescence, and ferredoxin (Figure 4b). The expression of some genes involved in cell cycle regulation, such as cell division control and regulator of chromosome condensation (RCC1), was also repressed in *lrl1‐1* (Figure 4b). Notably, there were no upregulated transcripts involved in autophagy and programmed cell death observed in conjunction with P‐starvation, which is consistent with the previous transcriptomics results (Bajhaiya *et al*., 2016). The global change in the transcriptome under P‐starvation in *C. reinhardtii* has also been observed in other species, such as *Nannnochloropsis oceanica*, with similar responses in the upregulation of genes related to P‐acquisition, phospholipid recycling, and, particularly, DGTS and SQDG synthesis (Mühlroth *et al*., 2017). The overall transcriptomics results suggested that, in addition to regulation by PSR1, responses during P‐starvation are also being regulated by LRL1, particularly during the later stage of starvation.

### Porphyrin‐related metabolites accumulate differently in *lrl1‐1* during early P‐starvation

Some MYB TFs regulate the accumulation of secondary metabolites in plants (Ambawat *et al*., 2013). A metabolome analysis was conducted by liquid chromatography−tandem mass spectrometry (LC‐MS/MS) that identified notable differences after 2 days under P‐replete and P‐depleted conditions. The resulting PCA plot showed that metabolite variances differed between WT and *lrl1‐1* after 2 days in culture*,* suggesting that LRL1 regulates secondary metabolism during the early transition to P‐starvation (Figure S11a). No observable differences were detected after 4 and 8 days in culture. Interestingly, metabolites that were annotated as porphyrin degradation‐related metabolites increased in *lrl1‐1* under P‐depleted conditions. Pheophorbide *a* oxygenase (PaO) converts pheophorbide *a* into red chlorophyll catabolite (RCC) and is involved in the chlorophyll degradation pathway (Pružinská *et al*., 2003). As shown in Figure S11(b), there was less pheophorbide *a* in *lrl1‐1* than in WT after 4 days under P‐depleted conditions, whereas after 8 days the accumulation was similar between WT and *lrl1‐1*. RCC is one of the last products in the chlorophyll degradation pathway (Christ and Hörtensteiner, 2014). Throughout the time course, its accumulation tended to be increased in *lrl1‐1* under P‐starvation relative to WT (Figure S11b). The overall result of chlorophyll degradation is consistent with the lighter‐green phenotypic trait observed in *lrl1‐1* under P‐deficient conditions, in addition to the lower number of cells in culture (Figure 3b,d). As expected, the RNA‐seq results also showed a similar trend of an increase in the expression of some PaO‐like genes in *lrl1‐1* as compared with control after 2 days of P‐starvation (Data S2). RNA‐seq data also indicated that after 4 days of growth under P‐deficient conditions, the expression of chlorophyll degradation‐related genes was decreased in *lrl1‐1* (Data S2), suggesting a response to prevent further chlorophyll degradation and maintain growth under P‐starvation. A similar trend was noted for the chlorophyll‐related biosynthesis genes, some of which were repressed after 2 days in culture relative to 4 days under P‐depleted conditions (Data S3).

### Protein−protein interaction through the LRL1−CrbHLH2−CrTTG1 complex transactivates the *CrSQD2‐2* promoter in tobacco leaves

**Figure 6:**
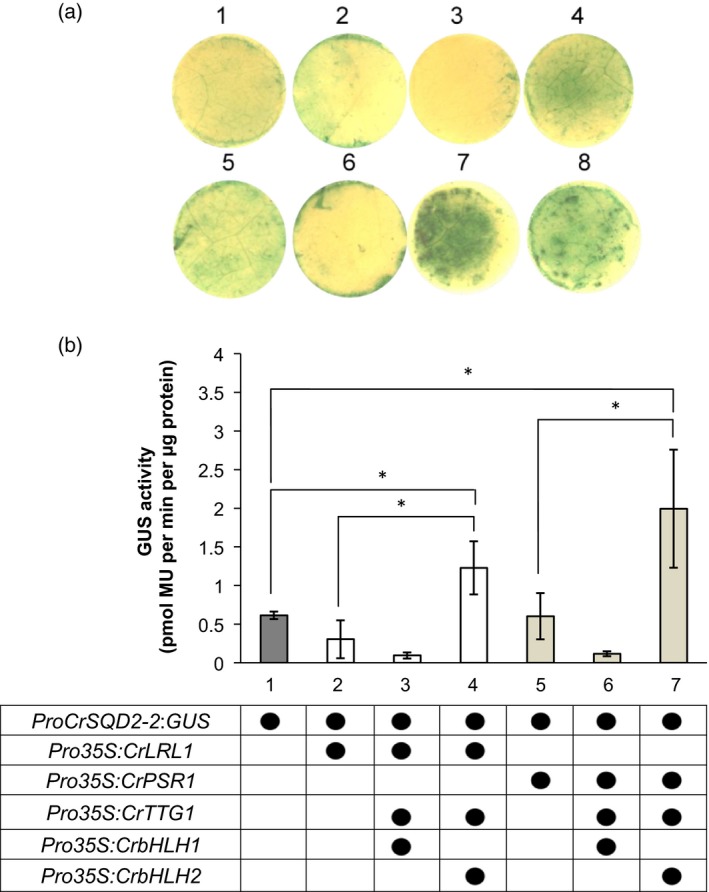
Transactivation of *ProCrSQD2‐2:GUS* in agroinfiltrated *Nicotiana benthamiana* leaves. (a) Histochemical GUS assay was carried out in *N. benthamiana* leaves 3 days after infiltration with *A. tumefaciens* cells harboring (1) *ProCrSQD2‐2*:*GUS;*(2) *ProCrSQD2‐2*:*GUS*/*35S*:*LRL1*; (3)*ProCrSQD2‐2*:*GUS*/*Pro35S*:*LRL1*/*Pro35S*:*CrTTG1*/*Pro35S*:*CrbHLH1*; (4)*ProCrSQD2‐2*:*GUS*/*Pro35S*:*LRL1*/*Pro35S*:*CrTTG1*/*Pro35S*:*CrbHLH2*; (5) *ProCrSQD2‐2*:*GUS*/*Pro35S*:*PSR1*; (6) *ProCrSQD2‐2*:*GUS*/*Pro35S*:*PSR1*/*Pro35S*:*CrTTG1*/*Pro35S*:*CrbHLH1*; (7) *ProCrSQD2‐2:GUS*/*Pro35S*:*PSR1*/*Pro35S*:*CrTTG1*/*35S*:*CrbHLH2*; and (8) *ProAtSUR1*:*GUS*/*Pro35S*:*AtMYB28*. (b) A transient fluorometric assay of *N. benthamiana* leaves 3 days after infiltration with *A. tumefaciens*. GUS activity was normalized to the total protein content. Two‐tailed Student's *t*‐test was carried out; asterisk (*) indicates values that are significantly different (*P < *0.05).

In *A. thaliana*, MYB protein regulates many cellular processes, such as hormone signaling, the circadian clock, cell wall and trichome formation, and the formation of specialized metabolites through the MYB–bHLH−TTG1 complex (Heim, 2003; Xu *et al*., 2015). To examine the specificity of LRL1 in regulating SQD2 genes, we used a transient β‐glucuronidase (GUS) expression assay by agroinfiltration in *Nicotiana benthamiana* tobacco leaves. We first tested the feasibility of the TF promoter from the *C. reinhardtii* genes in the tobacco leaves and compared the histochemical GUS staining with previously characterized *A. thaliana MYB28* and its promoter target (Hirai *et al*., 2007). As shown in Figure 6(a), co‐infiltration of *AtMYB28* and an *AtSUR1* promoter−driven reporter resulted in a strong GUS signal, as shown by the blue color. This indicated that there is an interaction between the TF and the promoter in the tobacco system. Then, we initially tested the interaction between the *CrSQD2‐2* promoter with LRL1 and with PSR1. Infiltration of the *CrSQD2‐2* promoter itself resulted in weak activity, but there was no significant difference between infiltration of the promoter only and co‐infiltration with *PSR1* or *LRL1*and the promoter (Figure 6b). We hypothesized that there might be other algal protein(s) that cooperatively function with the TFs and that interactions with the other algal factor(s) might be needed for the appropriate function of LRL1 and PSR1 in the tobacco system. Based on the phylogenetic analysis, a TTG1 homolog and 10 bHLH homologs were found in *C. reinhardtii* (Figures S13 and S14). Two bHLH genes from different clades were chosen for additional co‐infiltration experiments. As expected, there was a significant increase in GUS activity when *CrTTG1* and *CrbHLH2* were co‐expressed with *PSR1 and LRL1* (Figure 6). The algal protein–protein interactions for LRL1/PSR1‐CrbHLH2‐CrTTG1 were thus highly specific for members of this complex and were also specific for its target promoter, whereas other promoter targets tested in this study showed only a slight increase from the basal level (Figure S12). Overall, the transactivation assay in tobacco indicated that the binding of LRL1 to the *CrSQD2‐2* promoter happened through protein–protein interactions with another regulatory protein and another TF. Meanwhile, protein–protein interactions failed to enhance the activation of other promoters, such as *CrSQD2‐1*,* CrPHT1*, and *LRL1* (Figure S12). LRL1 showed only basal GUS activity when infiltrated with *CrSQD2‐1* or *CrPHT1* (Figure S12a). Although both genes were downregulated in *lrl1‐1* based on the RNA‐seq data, it may be possible that other factors contribute to the regulation of these genes by LRL1 in the tobacco system. PSR1 also failed to strongly transactivate the *CrSQD2‐1*,* CrPHT1*, and *LRL1* promoters (Figure S12b). The protein–protein interaction with *CrTTG1* and *CrbHLH2* slightly enhanced GUS activity to the basal level. These results suggest that PSR1 may regulate those genes, but it requires another regulatory factor to enhance its activity.

## Discussion

Accumulation of TAG and SQDG is a notable response throughout photosynthetic organisms that affects lipid remodeling to adapt to P‐deficiency (Shimojima, 2011). RNA‐seq analysis indicated that adaptation to P‐starvation by upregulation of TAG synthesis and sulfolipid biosynthesis genes was repressed in *lrl1*. In agreement with the changes in the level of transcripts, TAG content and oil droplet formation were strongly impaired in *lrl1‐1* and *lrl1‐2*, together with suppression of membrane lipid remodeling in chloroplasts from PG to SQDG. It is well understood that to maintain Pi‐homeostasis in cells exposed to P‐depletion, the expression of genes encoding various phosphatases and those responsible for phospholipid degradation are increased (Nakamura, 2013). Synthesis of SQDG is also enhanced to compensate for phospholipid degradation and to maintain membrane integrity throughout oxygenic photosynthetic organisms (Hori *et al*., 2016). The RNA‐seq comparison and confirmation by real‐time PCR suggested that the P‐starvation response, including SQDG synthesis is suppressed in *lrl1‐1*. In fact, the molar ratio of SQDG was decreased along with suppression of the decrease in PG, a major phospholipid in chloroplast membranes. Consistent with this event, chlorophyll degradation was relatively increased in *lrl1‐1* during the early stage of P‐starvation. Thus, our overall results corroborate the possibility that LRL1 regulates these events at the same time during the response to P‐depletion.

TAG accumulates during the later stage of growth, that is during the stationary stage to store excess energy as a carbon source. *CrDGTT1* is an essential cue in N‐depletion that predicted the causality of high oil accumulation. During P‐depletion, *CrDGTT1* expression was upregulated, similar to its expression during N‐ or S‐depletion (Iwai *et al*., 2014; Sato *et al*., 2014). Real‐time PCR revealed that *CrDGTT1* expression was more suppressed at 4 days after P‐starvation. Suppression of *MLDP* and *SQD2* expression was observed earlier than suppression of *CrDGTT1* by 1 day after P‐depletion in *lrl1*. Therefore, it is still uncertain whether a decrease in TAG synthesis is also a primary effect of the LRL1 mutation under P‐depleted conditions.

Another form of carbon storage is the accumulation of starch in chloroplasts in P‐depleted cells. Starch synthesis was also slightly affected based on both the expression of the starch synthase 3 (*SS3*) gene and the accumulation of starch itself in *lrl1‐1* under P‐depletion. TAG accumulation, however, was much more dramatically affected in *lrl1*, even though it is a later‐occurring event during P‐depletion. This rather suggests that lipid remodeling in membranes and TAG accumulation are relatively primary events that are regulated by LRL1 under P‐depletion.

As described above, TAG accumulation under P‐starved conditions became more obvious during the later stages of growth, specifically when cells enter stationary phase. Physiological changes in P‐starved cells occur at a slower rate as compared with N‐starved or S‐starved cells (Schmollinger *et al*., 2014), and thus we carried out a co‐expression analysis based on the timing such that the cells had almost reached the state for metabolite accumulation. Repressing LRL1 also affected cell size under both P‐replete and P‐depleted conditions in *C. reinhardtii*. The *A. thaliana* TF MYB64 acts redundantly with AtMYB119 in regulating cell division and cellularization‐differentiation during female gametophyte development (Rabiger and Drews, 2013). The cellularization‐differentiation transition involves several regulatory processes related to cell growth, the cell cycle, and cellular differentiation. One of the regulators involved in cell cycle progression is retinoblastoma related (*rbr*) (Cross and Umen, 2015). In Arabidopsis, mutations affecting this gene result in additional nuclear divisions during female gametogenesis and led to defects in cell differentiation (Johnston *et al*., 2010). In *Chlamydomonas*, disruption of *MAT3*, which is a retinoblastoma gene ortholog, leads to significantly reduced cell size, an important aspect for *C. reinhardtii* during cell cycle progression (Olson *et al*., 2010). Our analysis of DEGs revealed that some cell cycle‐related genes were also affected in *lrl1‐1* when compared in P‐depleted and P‐replete conditions. *RCC1*, which affects the initiation of mitosis and chromatin decondensation, was one of these DEGs. As *lrl1* shows a smaller cell size, even under P‐replete conditions, it may be possible that LRL1 is a regulator of the cellularization‐differentiation transition in *C. reinhardtii* and thus controls lipid remodeling under P‐deficiency. However, it should be noted that any nutrient, including P, may be limiting during later growth stages under nutrient‐sufficient conditions.

In any case, synergism between the role of LRL1 in the cellularization−differentiation transition and its role in cell cycle regulation might occur either during a later stage of growth under nutrient‐sufficient conditions or during adaptation to nutrient‐deficient conditions.

In this study, we provide evidence that co‐expression analysis is a reliable tool for conducting a reverse genetics study to clarify gene function in microalgae, as well as in plants. Previously, an approach that relied on proteomics data successfully predicted that a TF, ROC40, which was known to be involved in circadian clock regulation in *C. reinhardtii*, is also involved in early events during short‐term exposure to N‐starvation in *Chlorella* UTEX29 (Goncalves *et al*., 2016). A master regulator of the P‐starvation response, PHR1, has been found in *A. thaliana*. PSR1, a homolog of PHR1*,* was identified in *C. reinhardtii*, and this TF regulates carbon reallocation under P‐starvation (Rubio *et al*., 2001). We speculate that LRL1 functions during a slightly later stage in the response to P‐deficiency. PSR1 regulates early, specific P‐starvation responses, such as P‐acquisition from extracellular and intracellular sources, as well as the activation of extracellular phosphatase activity, and thus determines the long‐term survival of the cell during P‐starvation (Shimogawara *et al*., 1999; Wykoff *et al*., 1999). Moreover, increased transcript levels of genes related to P‐scavenging and ‐recycling, such as PHOX, PTA, and PTB, appeared to require PSR1 when induced in P‐starvation. Several transcripts, such as *PTB4* and *PTB9*, were differentially regulated in *lrl1* after 2 days of P‐deficiency (Figure S10). Several P‐transporters and phosphodiesterases were also repressed in *lrl1* during the earlier stage of P‐starvation, thus indicating the possibility of a cooperative regulation between PSR1 and LRL1 in response to P‐starvation.

After 24 h of P‐starvation, cell growth is impaired in a *psr1* mutant along with the increase in *PSR1* transcripts in WT cells (Wykoff *et al*., 1999). In *lrl1* mutants, cell growth was obviously repressed after 4 days of P‐starvation, which coincides with the time point at which *LRL1* transcripts were highly accumulated in WT cells. Similar to *PSR1*,* LRL1* was also expressed under nutrient‐replete conditions, but with the possible role of specifically regulating the cell cycle to control cell size. The affected cell cycle‐related genes might determine the fate of cells when undergoing the rapid alternating cycling through S phase and mitosis to produce daughter cells under normal conditions and thus could continue to affect growth during P‐depletion. Interestingly, overexpression of PSR1 in the CC‐125 background obviously changes the accumulation of lipid and starch by increasing the transcript level of genes related to starch and lipid synthesis (Bajhaiya *et al*., 2016). Moreover, increased lipid accumulation in PSR1‐overexpressing lines occurs under nutrient‐replete conditions, showing the robustness of this TF in upregulating lipid accumulation without severely affecting cell growth (Ngan *et al*., 2015).

**Figure 7:**
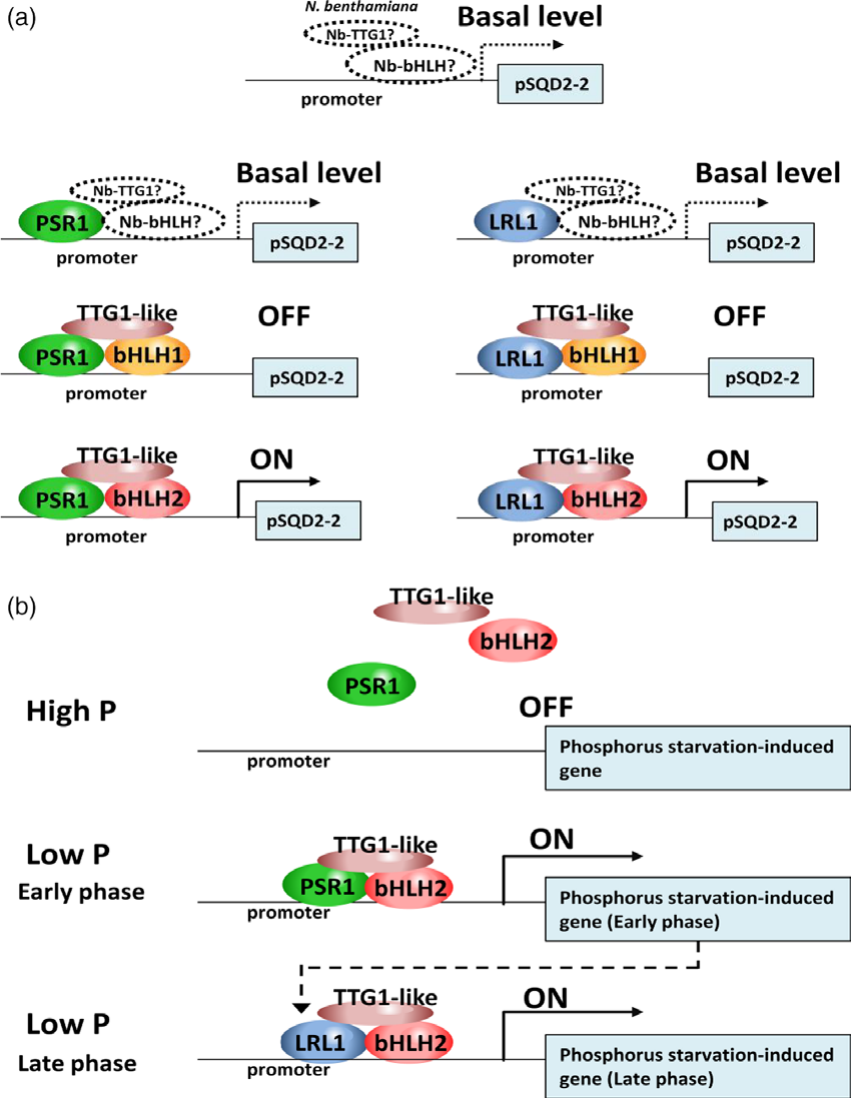
Proposed working model for *CrSQD2‐2* transcript activation in *N. benthamiana* and *C. reinhardtii*. (a) Working model of *CrSQ2‐2* transcript activation in *N. benthamiana*. Native TTG1‐like and other native TFs from *N. benthamiana* are initially involved in the transactivation of the *CrSQD2‐2* promoter in tobacco leaves. When *LRL1*,*PSR1*,* CrbHLHs*, and *CrTTG1* are expressed in the leaves, the protein−protein interactions determine the binding specificity to the *CrSQ2‐2* promoter region. (b) Working model of *CrSQ2‐2* transcript activation in *C. reinhardtii*. PSR1 as a master regulator of the phosphorus (P)‐starvation response was primarily induced to activate the transcription of genes that are inducible by P‐starvation. Its activation involves some protein–protein interactions with other regulatory factors and TFs, such as TTG1‐like and bHLH genes. The same mechanism is proposed for the activation of P‐starvation‐induced genes by *LRL1* during the prolonged phase of P‐starvation.

As noted above, two major lipid remodeling systems − TAG accumulation and SQDG synthesis − are commonly activated upon P‐deficiency. The promoter‐TF transient GUS expression in tobacco leaves suggested that LRL1 regulates the transcription of *CrSQD2‐2* through the interaction with other regulatory factors and TFs, and a similar mechanism also occurs for PSR1 (Figure 7a). It is not clear why a certain interaction with bHLH genes is required to induce the activation by MYB protein. In the case of R2R3‐MYBs that regulate flavonoid and glucosinolate (GSL) biosynthesis in *A. thaliana*, their function is also dependent on *bHLH* (Hernandez *et al*., 2004). R2R3‐MYBs are known to have low affinity for DNA, so the requirement to interact with bHLH is indispensable (Sainz *et al*., 1997). Overall, our findings suggest that LRL1 and PSR1 can bind and interact with the *CrSQD2‐2* promoter in tobacco, MYB determines the specificity to the promoter target, and TTG1 and bHLH are essential for regulating the transcriptional activation of the MYB‐encoding genes itself. As TTG1 has only one homolog and bHLH has only 10 homologs in *C. reinhardtii* (Figures S13 and S14), their specificity with respect to regulating certain transcripts may be low compared with the MYB and MYB‐related family proteins, which have 16 and 30 homologs in *C. reinhardtii*, respectively (Jin *et al*., 2017). These data also showed the complexity of transcriptional regulational mechanism under P‐starvation in *C. reinhardtii*. It is noted that even PSR1 as a master regulator of P‐starvation requires a specific factor to enhance its transcription activity, based on a transactivation assay in tobacco, indicating a subtle interaction with its target promoters. *SPX* might be involved in the regulational control of PSR1 (Liu *et al*., 2018), thus opening up a possibility for its involvement as an LRL1 transcriptional regulator.

Overall, LRL1 was involved in the regulatory mechanism during the later stage of P‐starvation in *C. reinhardtii* (Figure 7b) and may be indirectly regulated by PSR1, as its regulation might depend on P‐status, cell growth, and other factors. The possibility also remains that LRL1 is involved in the regulation of the cell cycle under nutrient‐replete and ‐depleted conditions, as a dataset in ALCOdb suggests that LRL1 is also upregulated under N‐starvation (Aoki *et al*., 2016). The secondary effect on cell cycle control might lead to the physiological impact of metabolite accumulation, such as TAG accumulation. In any case, our findings for LRL1 strongly suggest the presence of a tight connection between adaptations to nutrient starvation and cell cycle control in *C. reinhardtii* through the function of LRL1.

## Experimental procedures

### Co‐expression analysis

Co‐expression data used in this study were downloaded from ALCOdb (Cre‐R1‐15‐08) (Aoki *et al*., 2016). TFs co‐expressed with *CrDGTT1* in *C. reinhardtii* were identified based on the annotations of the best BLASTP hit gene in *A. thaliana*. Annotations for *A. thaliana* TFs were gathered from three TF databases: AGRIS (Palaniswamy *et al*., 2006), PlantTFDB (Jin *et al*., 2014), and PlnTFDB (Pérez‐Rodríguez *et al*., 2010).

### *C. reinhardtii* strains and culture conditions

The *C. reinhardtii* strain IAM‐C9 (CC‐408, wild‐type mt−) and *lrl1‐1* (886‐G12) were obtained from the Kyoto University *C. reinhardtii* mutant library (Yamano *et al*., 2015). Screening of *lrl1‐1* from the *Chlamydomonas* mutant library was performed according to Gonzalez‐Ballestar *et al*. (2011). Briefly, several primers spanning the upstream region to the 3′ end region of Cre03.g197100 were designed. The superpool library was used as the PCR template, and any amplified product from a primer pair that included a specific region of Cre03.g197100 and *AphVIII* were sequenced, and its positional tag was identified (Figure S3). Further DNA sequencing indicated that the *AphVIII*‐tag insertion was in the upstream region of Cre03.g197100, 115 bp upstream of the 5′ untranslated region (UTR), which is predicted to be the promoter region. *C. reinhardtii* strain CC‐4533 (*cw15* mt−) and *lrl1‐2* (LMJ.RY0402.178603) were obtained from a *Chlamydomonas* mutant library provided by the Jonikas laboratory (Li *et al*., 2016). The insertion site at the intron position was confirmed (Figure S3). Although most of the analysis in this work was carried out on *lrl1‐1*,* lrl1‐2* was also analyzed to confirm our results. Liquid cultures were grown in an Erlenmeyer flask mixotrophically in Tris−acetate−phosphate (TAP) medium (Gorman and Levine, 1965). For all cultures, cells were cultivated under continuous illumination at 20–40 μmol m^−2^ sec^−1^ and 100 rpm shaking at 25°C. To induce P‐starvation, mid‐log phase cells (3–5 × 10^6^ cells mL^−1^) were centrifuged at 2000 × ***g*** for 5 min and washed twice in a TAP medium. Potassium phosphate was replaced by 1.5 mm KCl in TAP −P medium (Quisel *et al*., 1996). Cells were initially adjusted to 1 × 10^5^ cells mL^−1^ on culture at day 0. Cell growth was monitored by measuring the optical density (OD) at 750 nm with a Shimadzu UV‐1800 spectrophotometer. Cell density was determined with a hemocytometer. Cell images were captured by digital camera from a fluorescence microscope (Nikon, Japan) with a ×40 objective lens. The area of individual cells was measured by image analyzing software (ImageJ).

### Phylogenetic analysis

MYB TF protein sequences were collected from *A. thaliana* and *C. reinhardtii* databases with the BLASTP program (Altschul *et al*., 1990) and were aligned using MAFFT v7.220 (Katoh and Standley, 2013). The trimAl v1.2 tool (Capella‐Gutiérrez *et al*., 2009) was used to remove any poorly conserved regions. The amino acid substitution model was calculated by Aminosan52 (Tanabe, 2011). Phylogenetic analyses were performed using the maximum likelihood method and the neighbor‐joining method in MEGA7 (Kumar *et al*., 2016) and with Bayesian analysis, which was performed with MrBayes 3.2.3 (Ronquist *et al*., 2012) for 1 000 000 generations. Every 500 generations were sampled, and the first 200 trees were discarded as burn‐in.

### RNA extraction and quantification of gene expression by real‐time qPCR

Total RNA was extracted using TRI Reagent (Sigma‐Aldrich, St. Louis, MO, USA) with a few modifications. Total RNA (500 ng) was used to synthesize the cDNA. First‐strand cDNA was synthesized with Superscript II (Invitrogen, St. Louis, MO, USA) by using oligo(dT)18 primer. Quantification of gene expression by real‐time qPCR was performed with the Thermal Cycler Dice Real‐Time System and Multiplate RQ software (TaKaRa, Otsu, Shiga, Japan). Gene expression was normalized relative to *CBLP* expression as ΔΔCt (Livak and Schmittgen, 2001). Primers used for the analyses are listed in Table S2.

### Nile red staining of neutral lipid droplets and confocal microscopy

Nile red stock solution was prepared as described (Greenspan *et al*., 1985). Living cells were stained with a Nile red solution diluted in acetone to a final concentration of 0.1 μg mL^−1^ and then were kept in the dark for ~10 min before observation with an LSM 780 confocal laser scanning microscope (Carl Zeiss, Oberkochen, Germany). A 488‐nm argon laser was used for excitation. Nile red signal was detected at 556–580 nm, whereas chlorophyll fluorescence was detected at 718–749 nm. Image processing was done by using the Zen software (Carl Zeiss).

### Lipid extraction and analysis

Total lipid extraction was carried out following the Bligh‐Dyer method (Bligh and Dyer, 1959). Neutral lipid fractions were separated by thin‐layer chromatography (TLC) (Merck, Darmstadt, Germany) with triolein as the control and developed with hexane/methanol/acetic acid (160:40:4, v/v/v). Polar lipid fractions were separated by two‐dimensional TLC with chloroform/methanol/water (130:50:8, v/v/v) as the first dimension followed by chloroform/methanol/isopropylamine/28% ammonia water (130:70:1:10, v/v/v/v) as the second dimension. Lipid spots were detected under UV light. Each lipid spot was scraped and extracted in 2.5% HCl in anhydrous methanol (Sigma**‐**Aldrich) at 85°C for 1 h. Pentadecanoic acid was added as the internal standard. The methanolysis‐derived fatty acid methyl esters (FAMEs) were extracted in hexane and analyzed by GC‐FID (GC‐2014, Shimadzu, Kyoto, Japan) that was equipped with a capillary column (BPX70, SGE Analytical Science, Ringwood, Australia). Supelco 37 Component FAME mix (Sigma‐Aldrich) was used as the FAME peak reference standard.

### Quantification of starch content

Starch was extracted and the amount was measured as described previously (Doebbe *et al*., 2010) using a starch assay kit (Roche Diagnostics GmbH, Mannheim/R‐Biopharm, Germany).

### Chlorophyll quantification

Chlorophyll extraction was carried out using 80% acetone. The resulting supernatant, which contains total chlorophyll, was isolated, and absorption was measured by a spectrophotometer at 646.6 and 663.6 nm (Porra *et al*., 1989).

### Plasmid construction

The vector backbone for the effector and GUS‐reporter construct was prepared by PCR amplification with primer ivec_pBI121_*Xho*I_fwd and ivec_pBI121_*Xho*I_rev, each of which contains an *Xho*I restriction enzyme site, using pBI121 vector as the template, followed by *Xho*I digestion and self‐ligation to obtain a pBI121 plasmid from which 2667 bp upstream of the CaMV*35S* promoter was removed. Approximately 1000 bp upstream of the transcription start site was determined to be the promoter region and was amplified by PCR using KOD Fx Neo (Toyobo, Osaka, Japan) with primers shown in Table S2. The sequence of *CrSQD2‐1* promoter region has been submitted to the DNA Data Bank of Japan (DDBJ)/EMBL/GenBank databases under accession number LC488724. The vector backbone for the promoter:GUS‐reporter fusion was linearized from the previously prepared pBI121 vector using primers GUS_F and pBI121_right_ivec. Coding sequences of *LRL1* and *PSR1* were amplified from cDNA templates and fused with the CaMV*35S* promoter from the same previously prepared pBI121 vector without the GUS sequence by using primer pBI121_left_ivec and ivec_35S_rev. These plasmids were all constructed using the *in vivo E. coli* cloning (iVEC) (Nozaki and Niki, 2019) or infusion system (Clontech, TaKaRa, Japan). The Gateway system (Thermo Fischer Scientific, Waltham, MA, USA) was used to construct *Pro35S:AtMYB28*. After performing first‐ and second‐round PCR using the primers shown in Table S2, *AtMYB28* was subcloned into pDONR/Zeo Vector by carrying out the BP reaction. Then, the LR reaction was carried out to obtain the final construct with pGWB2 as the vector backbone. The LR reaction was performed after restriction digestion of pGWB2 with *Xho*I. The iVEC system was used to prepare the *ProAtSUR1:GUS* construct.

### *Agrobacterium* infiltration of tobacco leaves

Each plasmid was transformed into *A. tumefaciens* prior to agroinfiltration into the tobacco leaves. Transformed *A. tumefaciens* was then grown overnight in liquid YEP medium at 30°C. Agroinfiltration was carried out as described (Yang *et al*., 2000). Tobacco (*Nicotiana benthamiana*) plants were grown for 6–7 weeks at 22°C under continuous white light (40–50 μmol m^−2^ sec^−1^).

### GUS activity determination

Tobacco leaves were collected 3 days after agroinfiltration and frozen in liquid nitrogen prior to extraction. In a 1.5‐mL tube, each leaf was ground in GUS extraction buffer (50 mm NaHPO_4_,pH 7.0; 10 mm 2‐mercaptoethanol; 10 mm Na_2_EDTA; 0.1% sodium lauryl sarcosine; 0.1% Triton X‐100). After centrifugation, supernatant was mixed with GUS assay solution (2 mm 4‐methylumbelliferyl‐d‐glucuronide in extraction buffer). The mixture was then incubated for 30 min at 37°C. A 100‐μl aliquot was removed immediately and added to 4.9 mL stop buffer (0.2 m sodium carbonate) to be used as the control. The rest of the mixture was incubated at 37°C for another 30 min. GUS activity was determined using a F‐2700 fluorescence spectrophotometer (Hitachi, Chiyoda, Japan), and protein concentration was determined with the Bradford reagent.

### Histochemical GUS staining

Leaf disks were vacuum filtrated for 10 min with staining solution containing 0.5 mg mL^−1^ 5‐bromo‐4‐chloro‐3‐indolyl‐β‐d‐glucuronic acid (Sigma‐Aldrich, USA) in 0.1 m sodium phosphate buffer (pH 7.3). Then the samples were incubated in the dark at 37°C until a blue‐indigo color appeared. The samples were then rinsed in 70% ethanol until the chlorophyll was removed.

### Metabolome analysis

*Chlamydomonas reinhardtii* cells were analyzed using an Agilent 1100 system (Agilent, Santa Clara, CA, USA) coupled to a Finnigan LTQ‐FT (Thermo Fisher Scientific). The detection and the alignment of the compound peaks in each sample were performed using an in‐house version of PowerGet software, which was modified for enabling batch processing (Sakurai *et al*., 2014). The metabolite candidates for the peaks were searched using the UC2 search function of MF Searcher tool (Sakurai *et al*., 2018) with a 5 ppm mass tolerance and the following compound databases: KEGG (Kanehisa *et al*., 2016), KNApSAcK (Afendi *et al*., 2012), HMDB (Wishart *et al*., 2013), LIPID MAPS (Fahy *et al*., 2009), and the flavonoid database in metabolomics.jp. The binary raw data from Xcalibur (.raw) and the experimental metadata for the samples are deposited at the Metabolonote website (http://metabolonote.kazusa.or.jp/SE195:/; http://webs2.kazusa.or.jp/data/nur/) (Ara *et al*., 2015).

### RNA‐seq and data analysis

The culture conditions and *C. reinhardtii* strains used for RNA‐seq are described in Table S1. The pelleted samples were disrupted by sonication in six volumes of RNA extraction buffer (0.8% SDS, 25 mm Tris−HCl (pH 7.6), 25 mm MgCl_2_, 25 mm KCl)/acid phenol (1:1, v/v). The aqueous phase was extracted three times with acid phenol/chloroform (1:1, v/v). Total RNA was precipitated by adding an equal volume of isopropanol. Next, RNA was purified using the RNeasy Plant Mini kit (Qiagen, Hilden, Germany). The library was prepared according to the protocol of the Illumina TruSeq RNA Sample Preparation kit v2. Sequencing was performed on the Illumina GAIIx platform. Reads were mapped to Joint Genome Institute (JGI) *C. reinhardtii* v5.5 (Merchant *et al*., 2007) using Bowtie2 ver2.2.5 (Langmead *et al*., 2009). The read counts were extracted from the output file and normalized using R package TCC ver1.2.0 (Sun *et al*., 2013).

## Accession numbers

Raw RNA‐seq data were deposited in the DDBJ Sequence Read Archive, under accession numbers DRX116072 to DRX116091 (Table S1).

## Author contributions

MS and HO designed and conceived the research. TO performed the co‐expression analysis. NAH performed most of the experiments with the help of YY‐O, MI, KS and KH. TY, MK, and HF generated the *Chlamydomonas reinhardtii* mutant library. KH conducted the RNA‐seq analysis. NS and KS provided technical support and analyzed the metabolome data. NAH and HO wrote the article. All authors commented and approved the article.

## Conflict of Interest

The authors declare no conflict of interest.

## Supporting information


**Figure S1.** Scatter plot and linear correlation of the expression profile of Cre12.g55750 (*CrDGTT1*) and Cre03.g197100 (*CrLRL1*).
**Figure S2.** Phylogenetic analysis of MYB proteins across the land plants and algae using the maximum likelihood method.
**Figure S3.** Molecular characterization of *lrl1‐1* and *lrl1‐2* mutants.
**Figure S4.** Analysis of fatty acid attached to triacylglycerol (TAG) extracted from mixotrophic normal and P‐starved conditions.
**Figure S5.** Fatty acid analysis of the membrane lipids extracted from mixotrophic P‐replete and P‐depleted conditions of C9 and *lrl1‐1* at day 8 in culture.
**Figure S6.** Hierarchical clustering and heatmap of the RNA‐seq data with 197 transcription factors in *C. reinhardtii*.
**Figure S7.** Principal component analysis (PCA) of 11 702 genes in the 20 RNA‐seq samples.
**Figure S8.** Transcriptome analysis of control and *lrl1‐1*under P‐replete and P‐depleted conditions.
**Figure S9.** Time course of real‐time qPCR for *CrSQD2*,* CrDGTT1*, and *MLDP* in WT, *lrl1‐1* and *lrl1‐2* under P‐depleted conditions.
**Figure S10.** Time course of quantitative real‐time PCR for *PTB2* and *PTB9* in WT and *lrl1‐1* under P‐depleted conditions.
**Figure S11.** Secondary metabolome analysis of control and *lrl1‐1* under P‐replete and P‐depleted conditions.
**Figure S12.** Transient GUS expression assay of different promoter targets and LRL1/PSR1 in *A. tumefaciens*‐infiltrated leaves.
**Figure S13**. Phylogenetic tree of some bHLH proteins in *A. thaliana* and *C. reinhardtii* as determined by RAxML with the LG model +G.
**Figure S14.** Phylogenetic tree of some TTG1‐like proteins across the land plants and algae as determined by RAxML with the LG model +G +F.

**Table S1.** List of RNA‐seq samples analyzed in this study.
**Table S2.** Primers used in this study.

**Data S1.** List of *Chlamydomonas reinhardtii* transcription factors (TFs) that resulted from co‐expression analysis with *CrDGAT2* (*CrDGTT1*).

**Data S2.** Normalized read count for genes related to chlorophyll synthesis, chlorophyll degradation, and lipid synthesis.

**Data S3.** Differentially expressed genes (DEGs) of control versus *lrl1‐1* under P‐replete and P‐depleted condition.
