# Supplementary material for: Lipid remodeling regulator 1 (LRL1) is differently involved in the phosphorus‐depletion response from PSR1 in Chlamydomonas reinhardtii
Source: Plant J. 2019 Aug 23;100(3):610–26. doi: 10.1111/tpj.14473 (PMC6899820; doi:10.1111/tpj.14473)
Supplement: Supplementary file 1 — Figure S1. Scatter plot and linear correlation of the expression profile of Cre12.g55750 (CrDGTT1) and Cre03.g197100 (CrLRL1). Figure S2. Phylogenetic analysis of MYB proteins across the land plants and algae using the maximum likelihood method. Figure S3. Molecular characterization of lrl1‐1 and lrl1‐2 mutants. Figure S4. Analysis of fatty acid attached to triacylglycerol (TAG) extracted from mixotrophic normal and P‐starved conditions. Figure S5. Fatty acid analysis of the membrane lipids extracted from mixotrophic P‐replete and P‐depleted conditions of C9 and lrl1‐1 at day 8 in culture. Figure S6. Hierarchical clustering and heatmap of the RNA‐seq data with 197 transcription factors in C. reinhardtii. Figure S7. Principal component analysis (PCA) of 11 702 genes in the 20 RNA‐seq samples. Figure S8. Transcriptome analysis of control and lrl1‐1under P‐replete and P‐depleted conditions. Figure S9. Time course of real‐time qPCR for CrSQD2, CrDGTT1, and MLDP in WT, lrl1‐1 and lrl1‐2 under P‐depleted conditions. Figure S10. Time course of quantitative real‐time PCR for PTB2 and PTB9 in WT and lrl1‐1 under P‐depleted conditions. Figure S11. Secondary metabolome analysis of control and lrl1‐1 under P‐replete and P‐depleted conditions. Figure S12. Transient GUS expression assay of different promoter targets and LRL1/PSR1 in A. tumefaciens‐infiltrated leaves. Figure S13. Phylogenetic tree of some bHLH proteins in A. thaliana and C. reinhardtii as determined by RAxML with the LG model +G. Figure S14. Phylogenetic tree of some TTG1‐like proteins across the land plants and algae as determined by RAxML with the LG model +G +F. [file TPJ-100-610-s001.pdf]

Figure S1

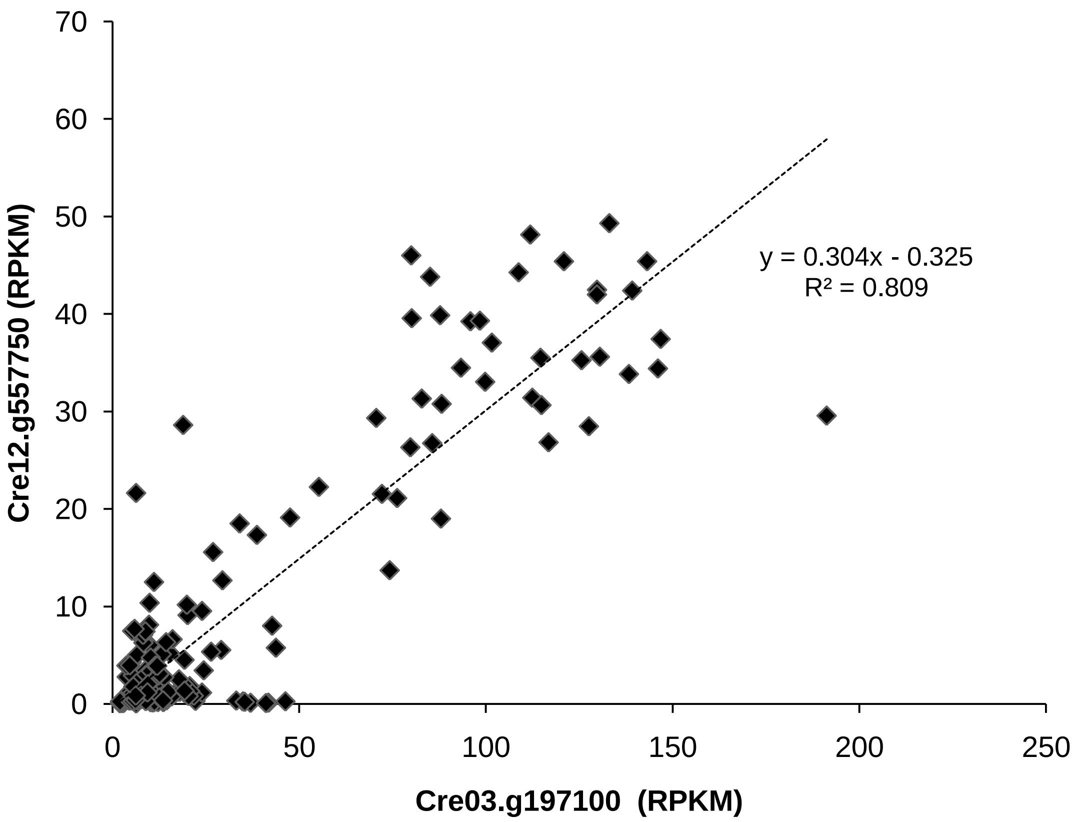

70
60
50
40
30
20
10
0
0
50
100
150
200
250
Cre12.g557750 (RPKM)
Cre03.g197100 (RPKM)
y = 0.304x - 0.325
R² = 0.809

Figure S2

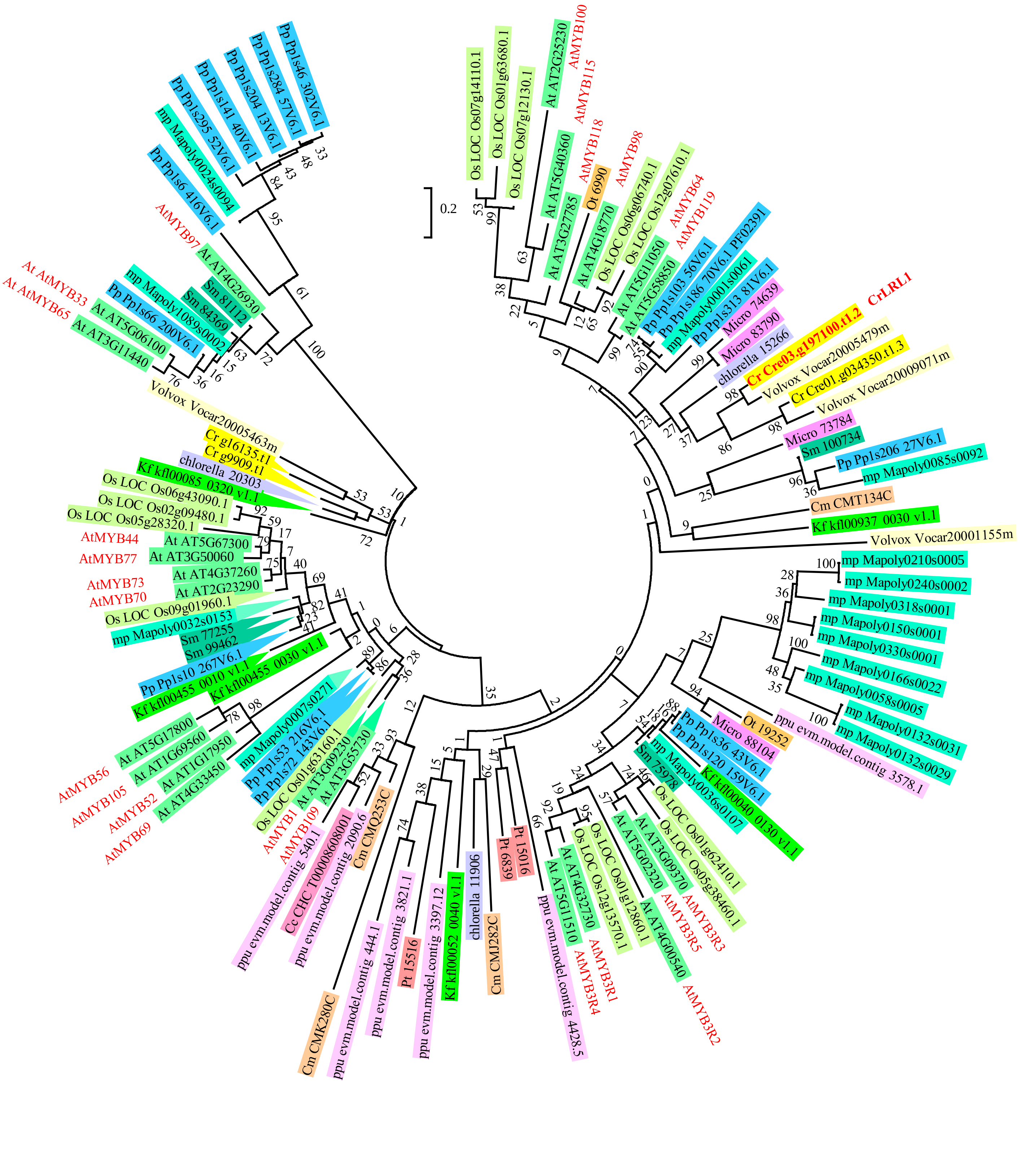

0.2
CrLRL1
Cr Cre03.g197100.t1.2
AtMYB100
AtMYB115
AtMYB118
AtMYB98
AtMYB64
AtMYB119
AtMYB97
At AtMYB33
At AtMYB65
AtMYB44
AtMYB77
AtMYB73
AtMYB70
AtMYB56
AtMYB105
AtMYB52
AtMYB69
AtMYB1
AtMYB109
AtMYB3R1
AtMYB3R4
AtMYB3R5
AtMYB3R3
AtMYB3R2

Figure S3

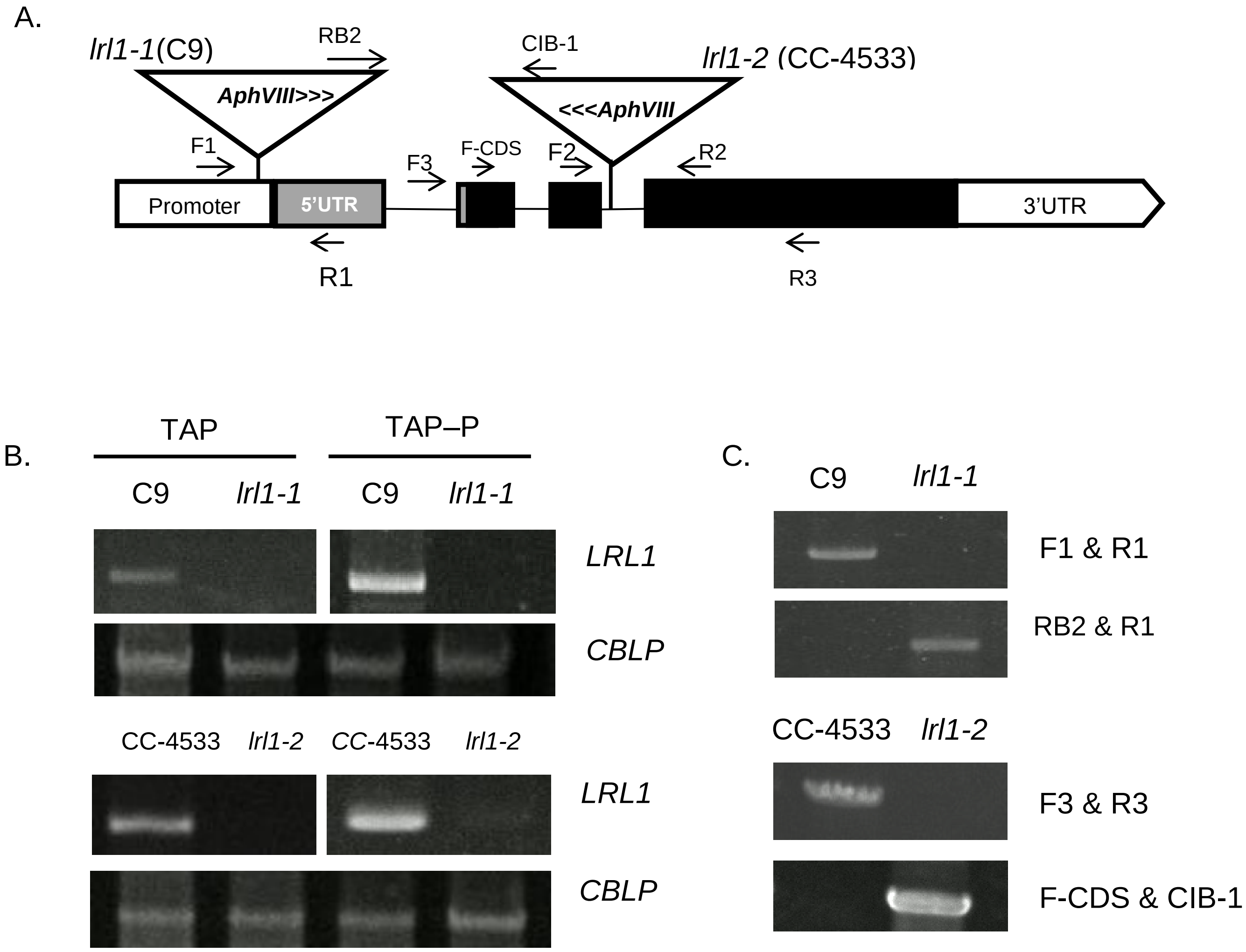
A.
lrl1-1(C9)
RB2
AphVIII>>>
F1
CIB-1
lrl1-2 (CC-4533)
<<<AphVIII
F3
F-CDS
F2
R2
Promoter
5'UTR
3'UTR
R1
R3
B.
TAP
TAP–P
C9
lrl1-1
C9
lrl1-1
LRL1
CBLP
CC-4533
lrl1-2
CC-4533
lrl1-2
LRL1
CBLP
C.
C9
lrl1-1
F1 & R1
RB2 & R1
CC-4533
lrl1-2
F3 & R3
F-CDS & CIB-1

Figure S4

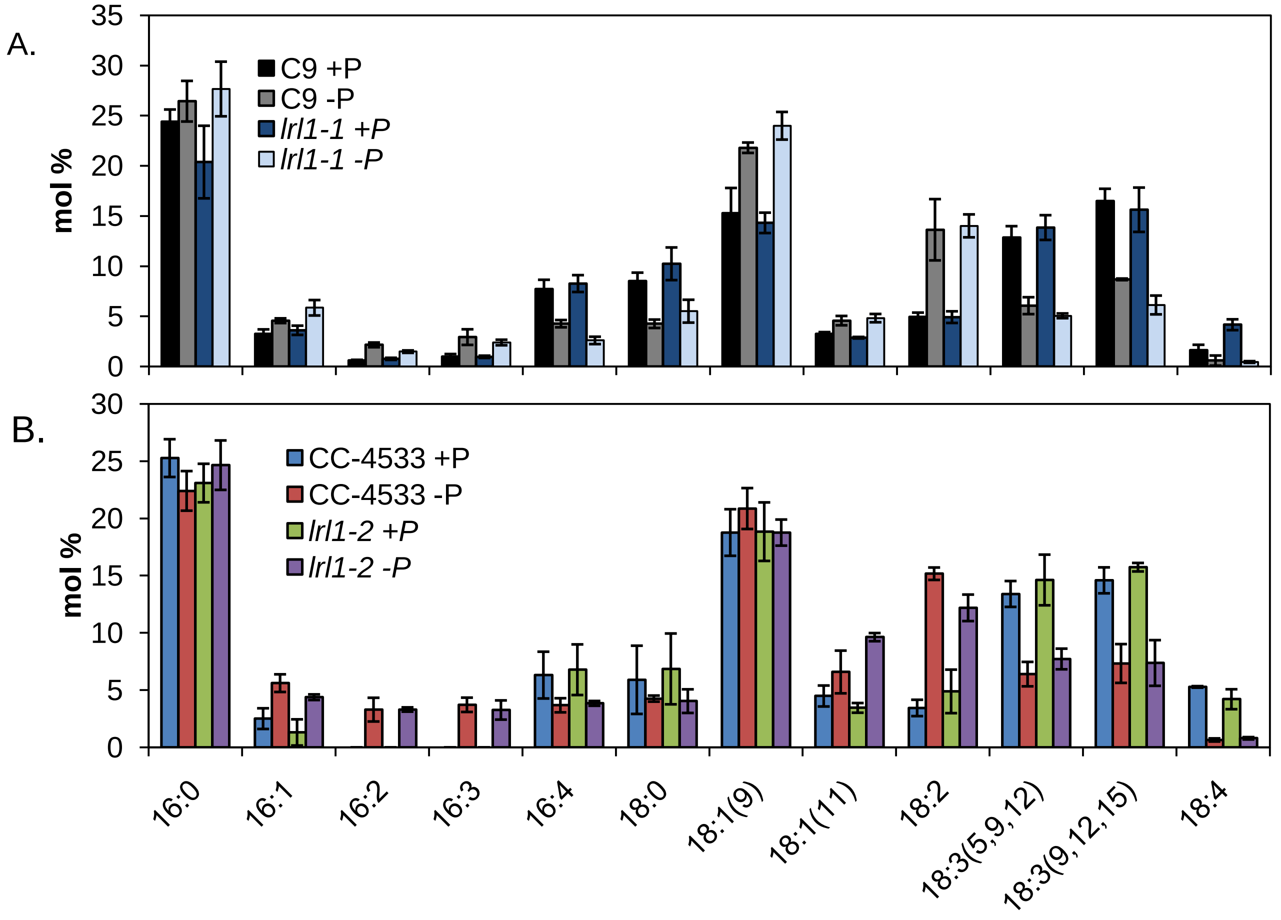
A.
C9 +P
C9 -P
lrl1-1 +P
lrl1-1 -P
mol %
B.
CC-4533 +P
CC-4533 -P
lrl1-2 +P
lrl1-2 -P
mol %
16:0
16:1
16:2
16:3
16:4
18:0
18:1(9)
18:1(11)
18:2
18:3(5,9,12)
18:3(9,12,15)
18:4

Figure S5

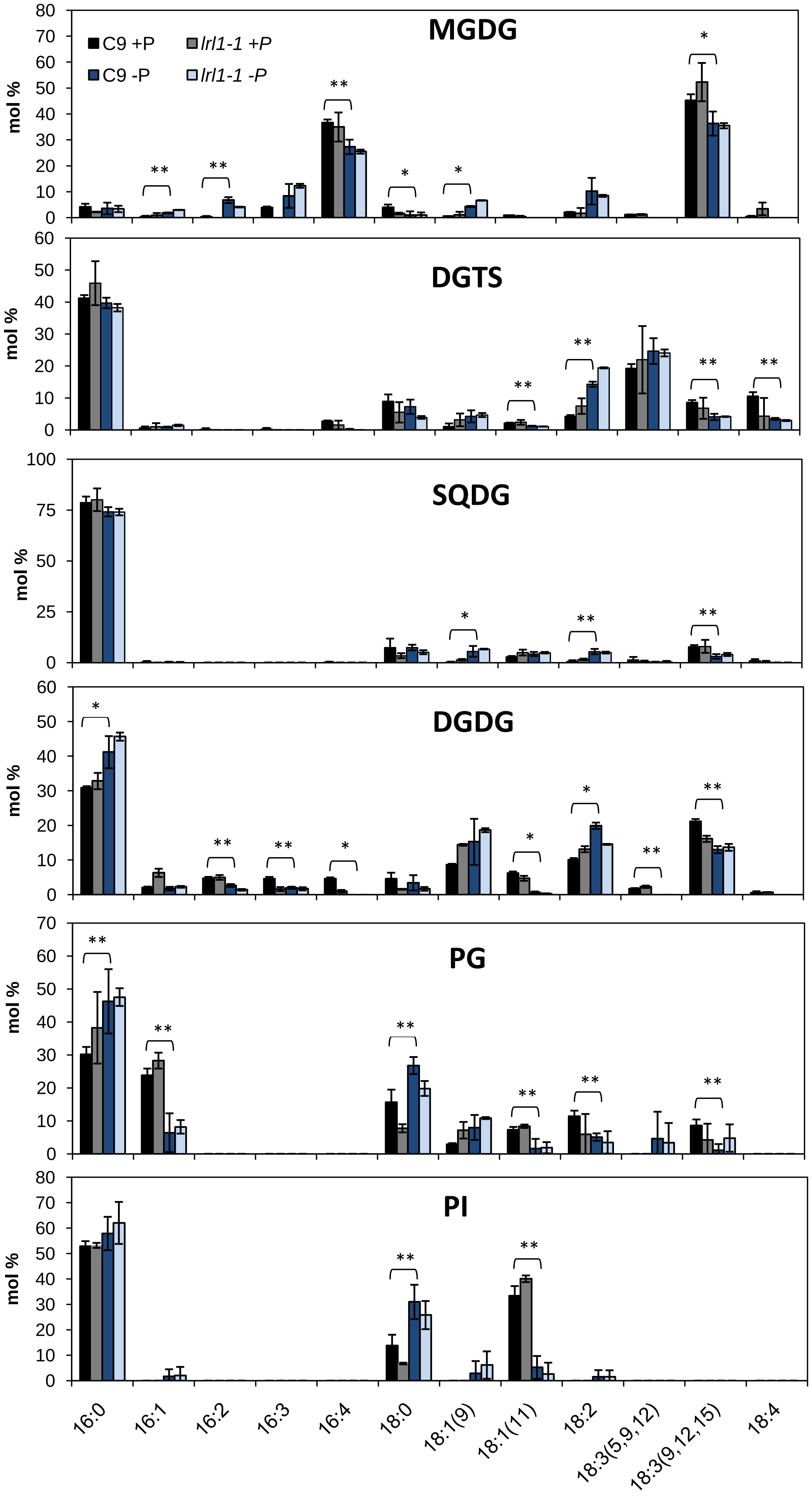
MGDG
DGTS
SQDG
DGDG
PG
PI
C9 +P
lrl1-1 +P
C9 -P
lrl1-1 -P
mol %
16:0
16:1
16:2
16:3
16:4
18:0
18:1(9)
18:1(11)
18:2
18:3(5,9,12)
18:3(9,12,15)
18:4

Figure S6

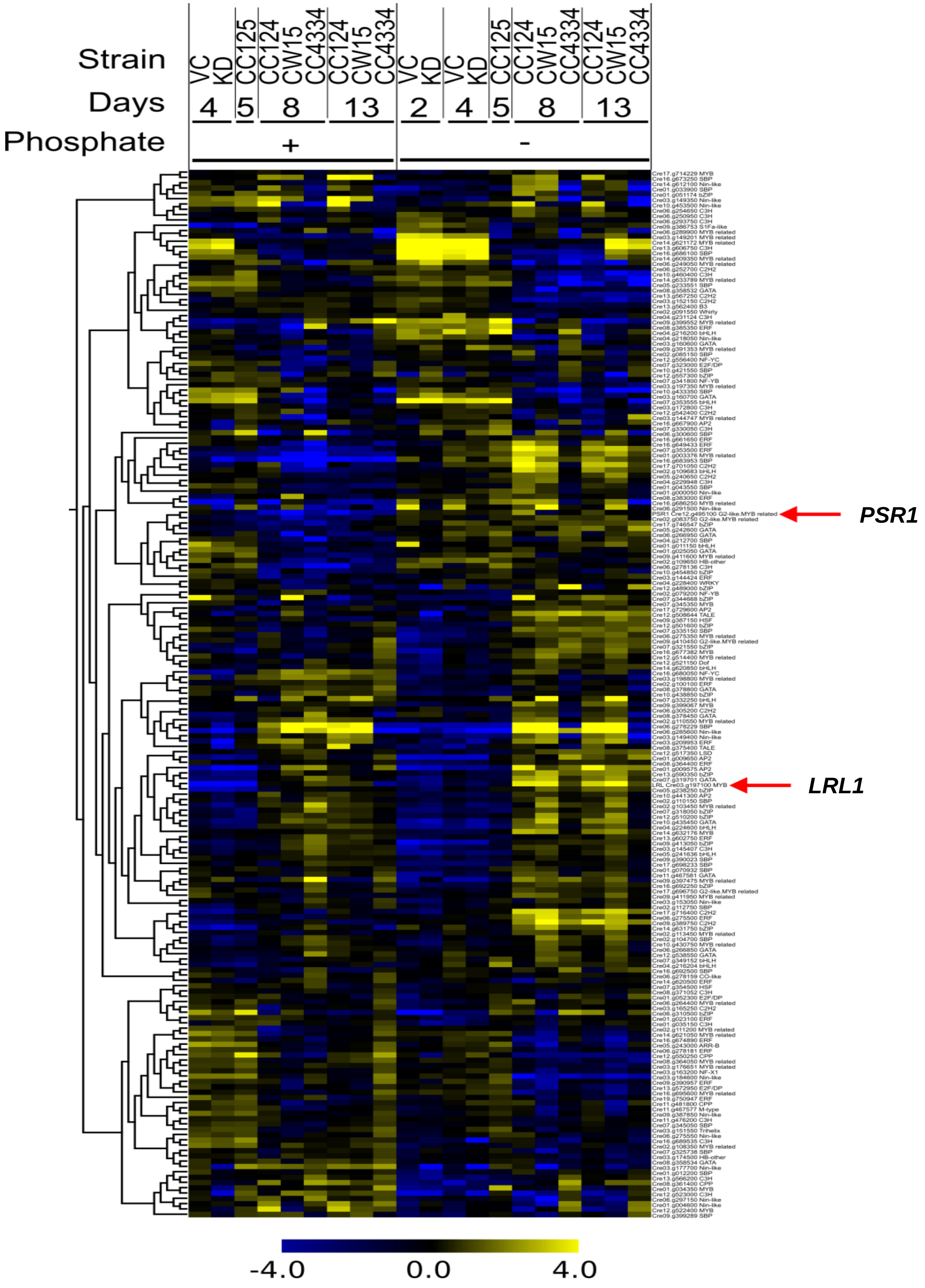

Strain
Days
Phosphate
VC
KD
CC125
CC124
CW15
CC4334
CC124
CW15
CC4334
VC
KD
VC
KD
CC125
CC124
CW15
CC4334
CC124
CW15
CC4334
4
5
8
13
2
4
5
8
13
+
-
PSR1
LRL1
-4.0
0.0
4.0

Figure S7

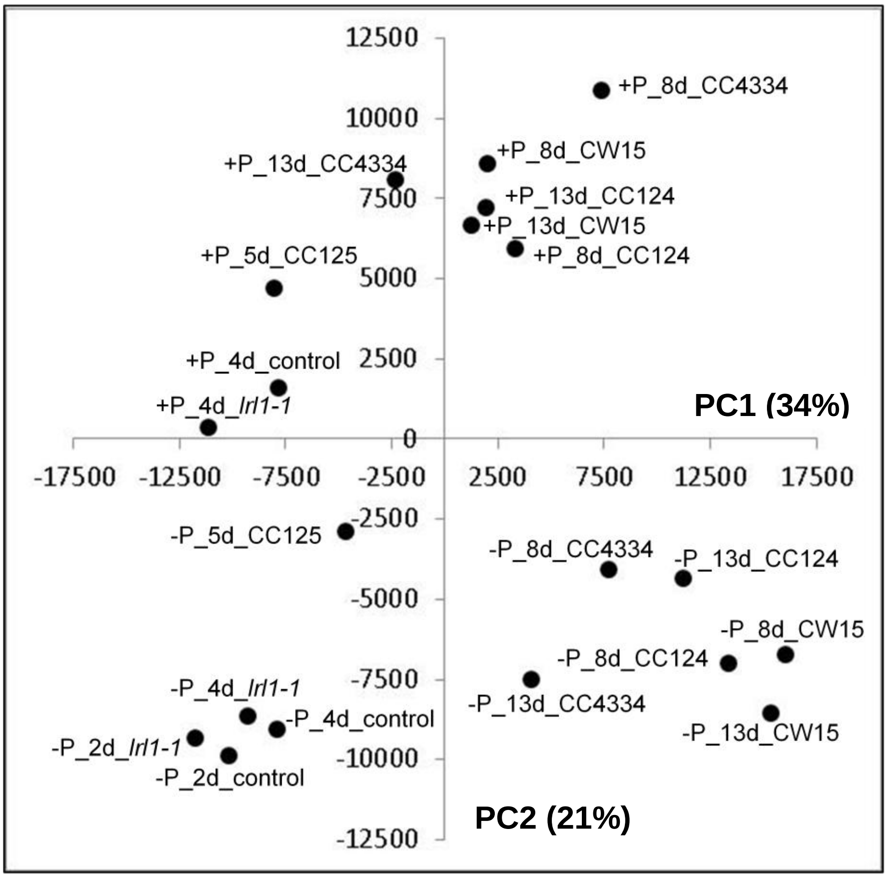

+P_8d_CC4334
+P_8d_CW15
+P_13d_CC4334
+P_13d_CC124
+P_13d_CW15
+P_8d_CC124
+P_5d_CC125
+P_4d_control
+P_4d_*lrl1-1*
PC1 (34%)
-P_5d_CC125
-P_8d_CC4334
-P_13d_CC124
-P_8d_CW15
-P_8d_CC124
-P_13d_CC4334
-P_13d_CW15
-P_4d_*lrl1-1*
-P_4d_control
-P_2d_*lrl1-1*
-P_2d_control
PC2 (21%)
12500
10000
7500
5000
2500
0
-2500
-5000
-7500
-10000
-12500
-17500
-12500
-7500
-2500
2500
7500
12500
17500

Figure S8

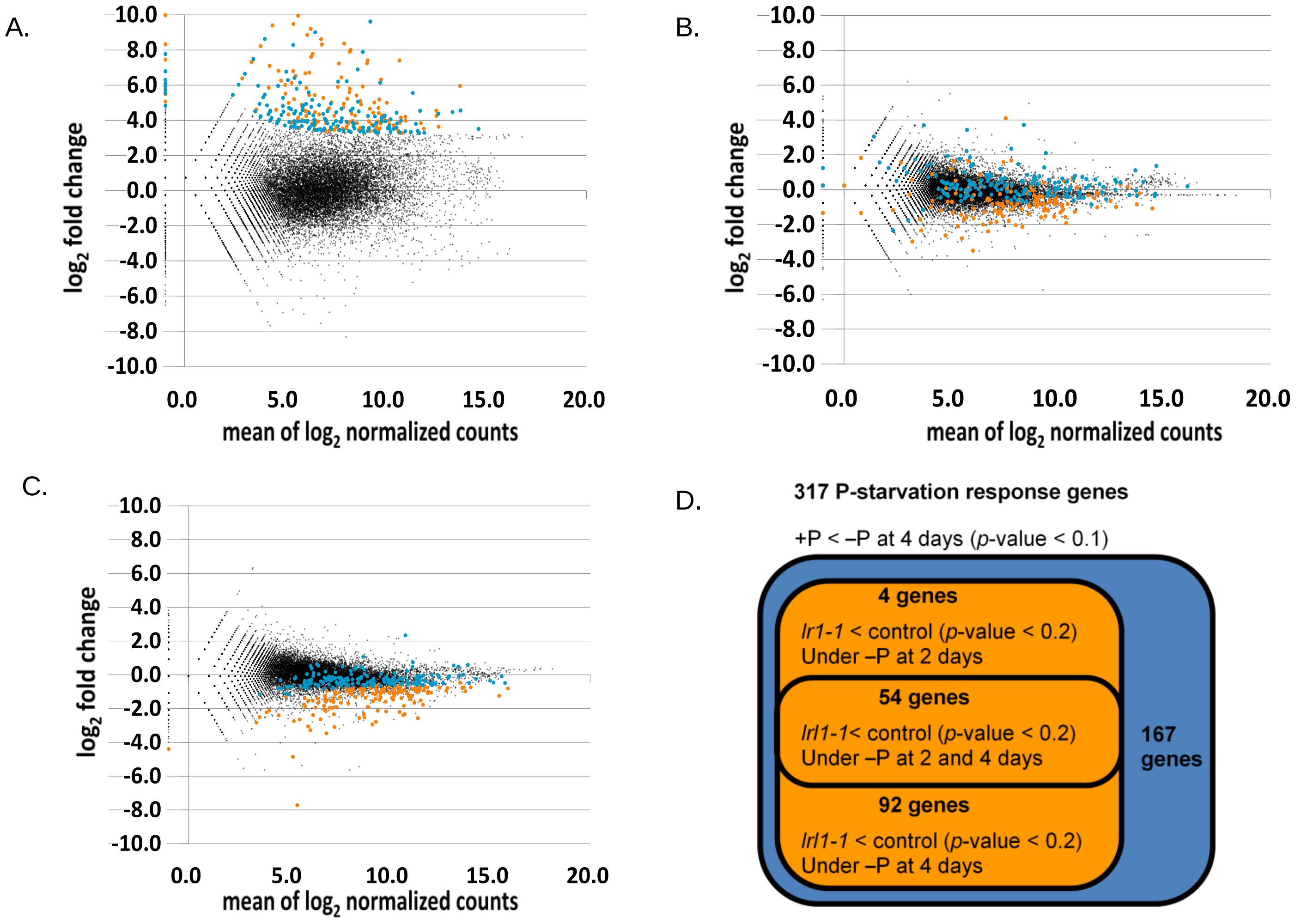
A.
B.
C.
log2 fold change
mean of log2 normalized counts
10.0
8.0
6.0
4.0
2.0
0.0
-2.0
-4.0
-6.0
-8.0
-10.0
0.0
5.0
10.0
15.0
20.0
D.
317 P-starvation response genes
+P < –P at 4 days (p-value < 0.1)
4 genes
lr1-1 < control (p-value < 0.2)
Under –P at 2 days
54 genes
lrl1-1< control (p-value < 0.2)
Under –P at 2 and 4 days
92 genes
lrl1-1 < control (p-value < 0.2)
Under –P at 4 days
167
genes

Figure S9

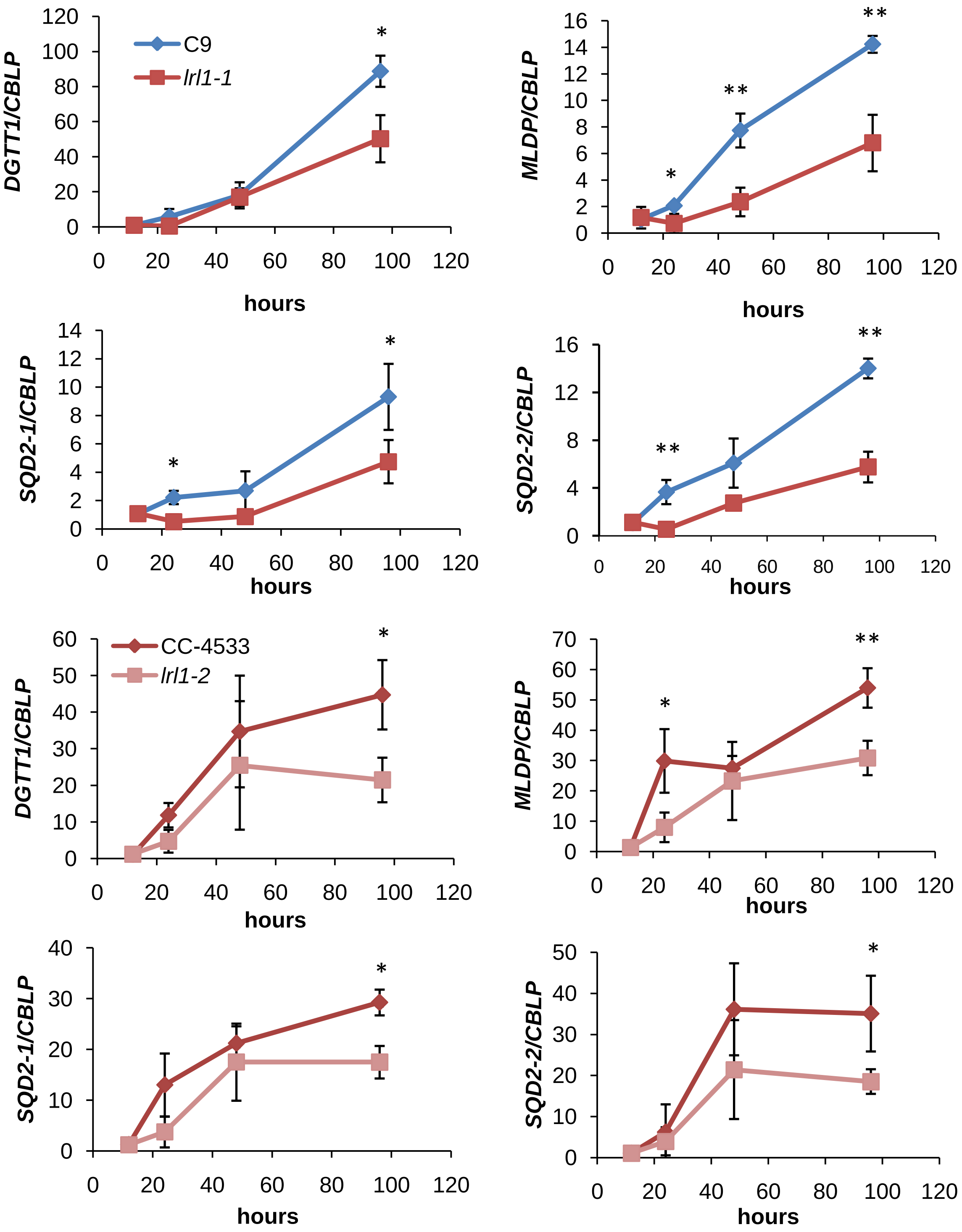
C9
lrl1-1
CC-4533
lrl1-2
DGTT1/CBLP
MLDP/CBLP
SQD2-1/CBLP
SQD2-2/CBLP
hours
*
**

Figure S10

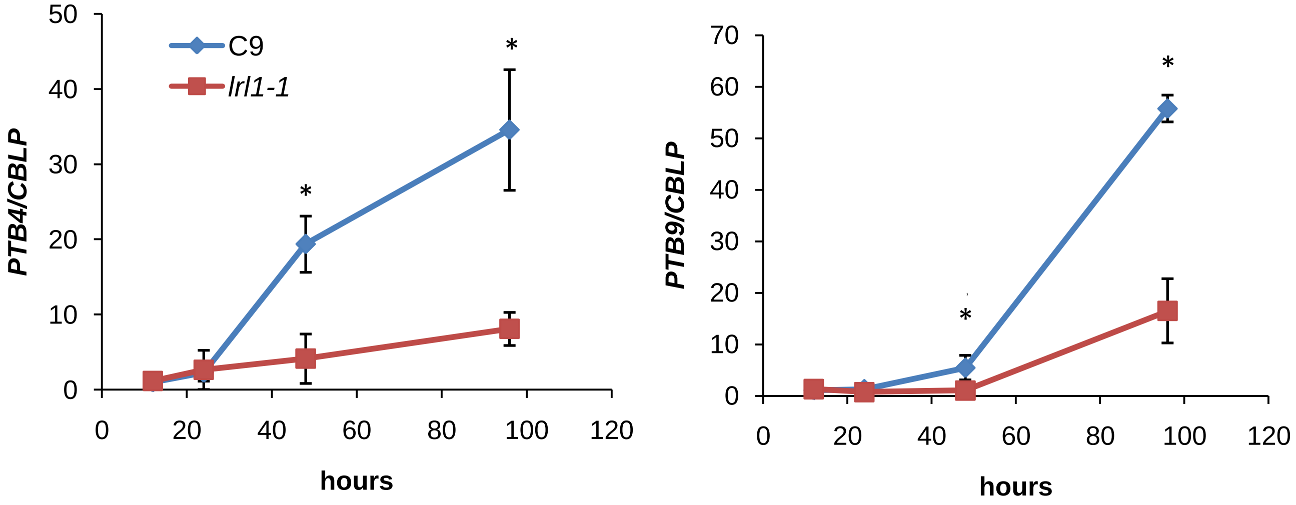

Figure S11

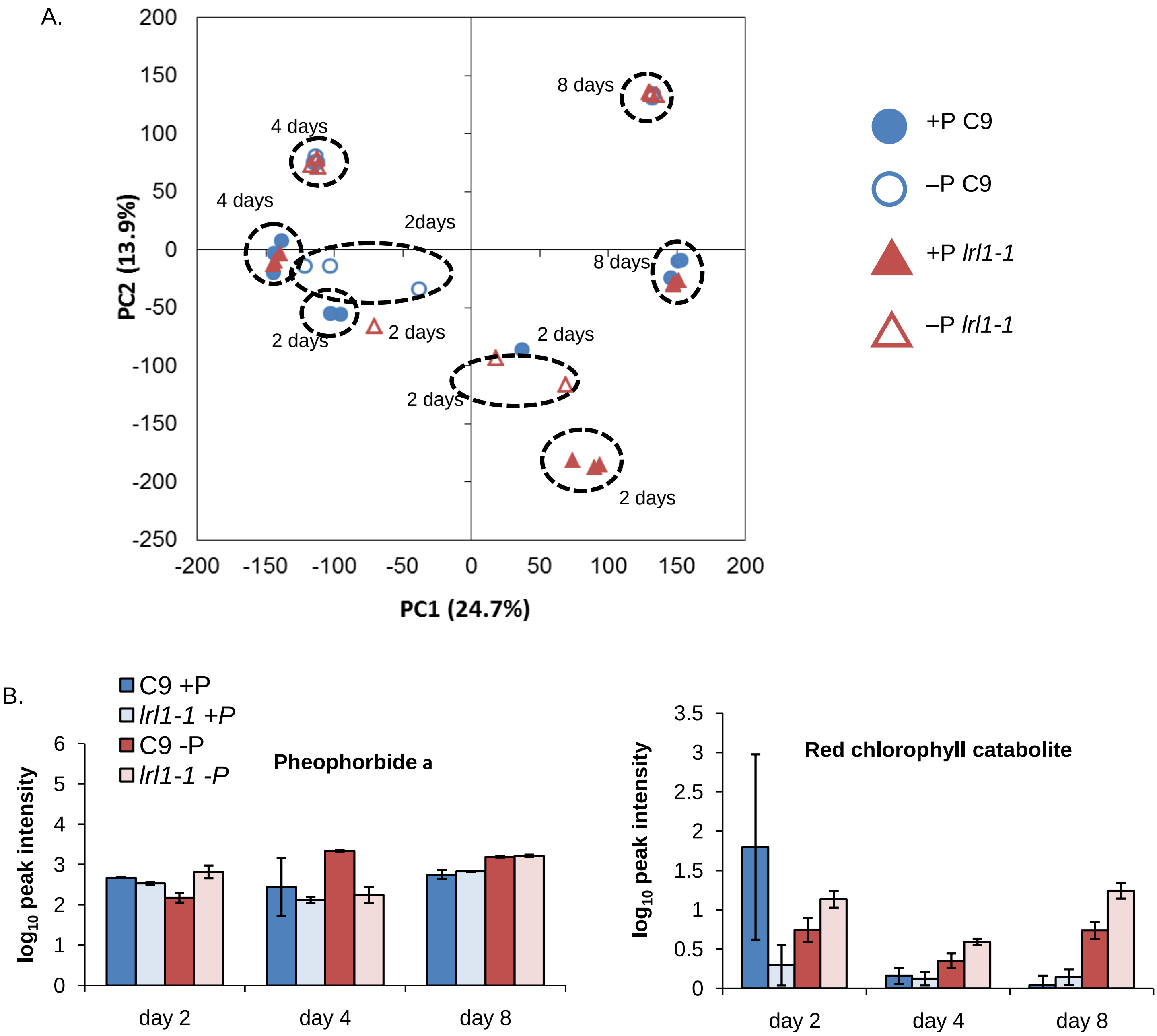
A.
PC1 (24.7%)
PC2 (13.9%)
4 days
4 days
2days
2 days
2 days
2 days
2 days
2 days
8 days
8 days
+P C9
–P C9
+P lrl1-1
–P lrl1-1
B.
C9 +P
lrl1-1 +P
C9 -P
lrl1-1 -P
Pheophorbide a
log10 peak intensity
day 2
day 4
day 8
Red chlorophyll catabolite
log10 peak intensity
day 2
day 4
day 8

Figure S12

A.

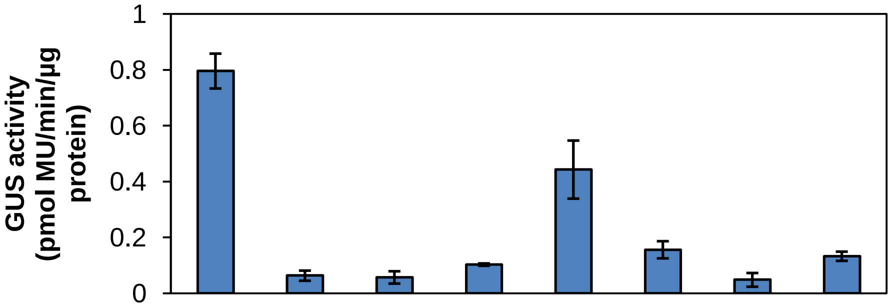

ProCrSQD2-1:GUS	●	●	●	●				
ProCrPHT1:GUS					●	●	●	●
Pro35S:CrLRL1		●	●	●		●	●	●
Pro35S:CrTTG1			●	●			●	●
Pro35S:CrbHLH1			●				●	
Pro35S:CrbHLH2				●				●

B.

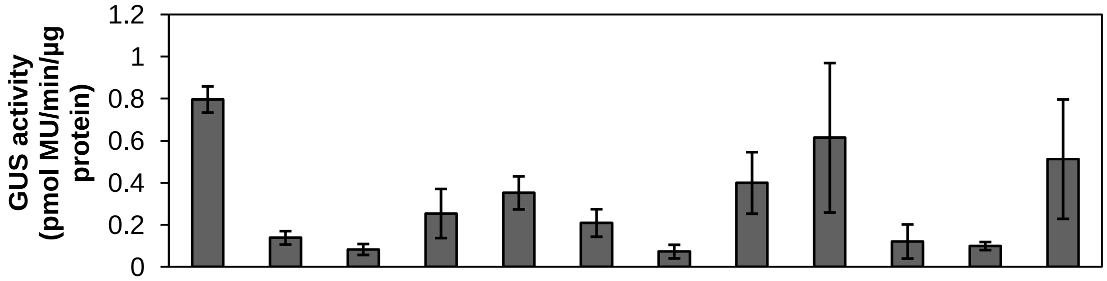

ProCrSQD2-1:GUS	●	●	●	●								
ProCrPHT1:GUS					●	●	●	●				
ProCrLRL1:GUS									●	●	●	●
Pro35S:CrPSR1		●	●	●		●	●	●		●	●	●
Pro35S:CrTTG1			●	●			●	●			●	●
Pro35S:CrbHLH1			●				●				●	
Pro35S:CrbHLH2				●				●				●

Figure S13

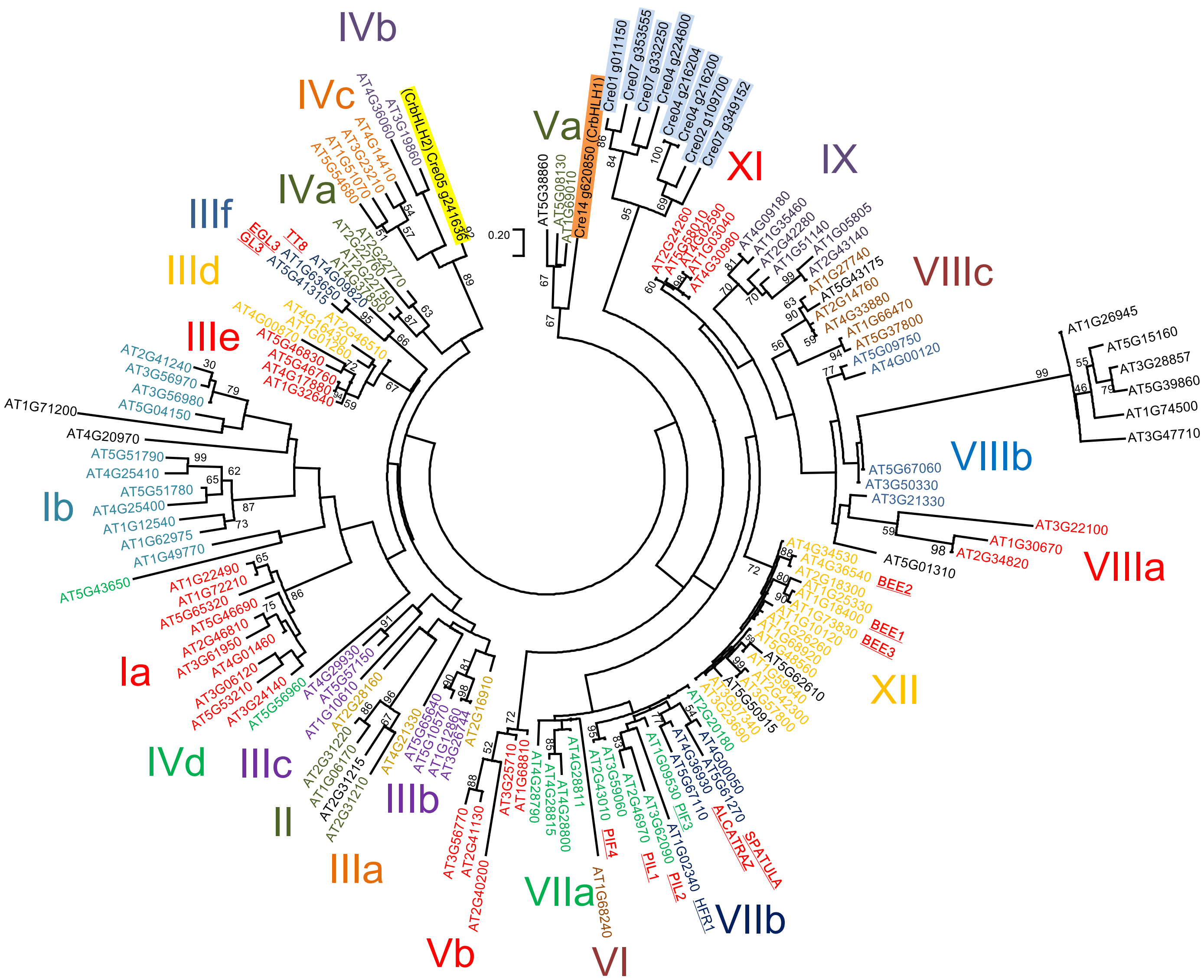

IVb
IVc
Va
XI
IX
IVa
IIIf
IIId
IIIe
VIIIc
VIIIb
VIIIa
Ib
Ia
XII
IVd
IIIc
II
IIIb
IIIa
VIIa
VIIb
Vb
VI
0.20
Cre14 g620850 (CrbHLH1)
(CrbHLH2) Cre05 g241636
Cre01 g011150
Cre07 g353555
Cre07 g332250
Cre04 g224600
Cre04 g216204
Cre04 g216200
Cre02 g109700
Cre07 g349152
BEE2
BEE1
BEE3
PIF4
PIF3
PIL1
PIL2
HFR1
ALCATRAZ
SPATULA
GL3
EGL3
TT8

Figure S14

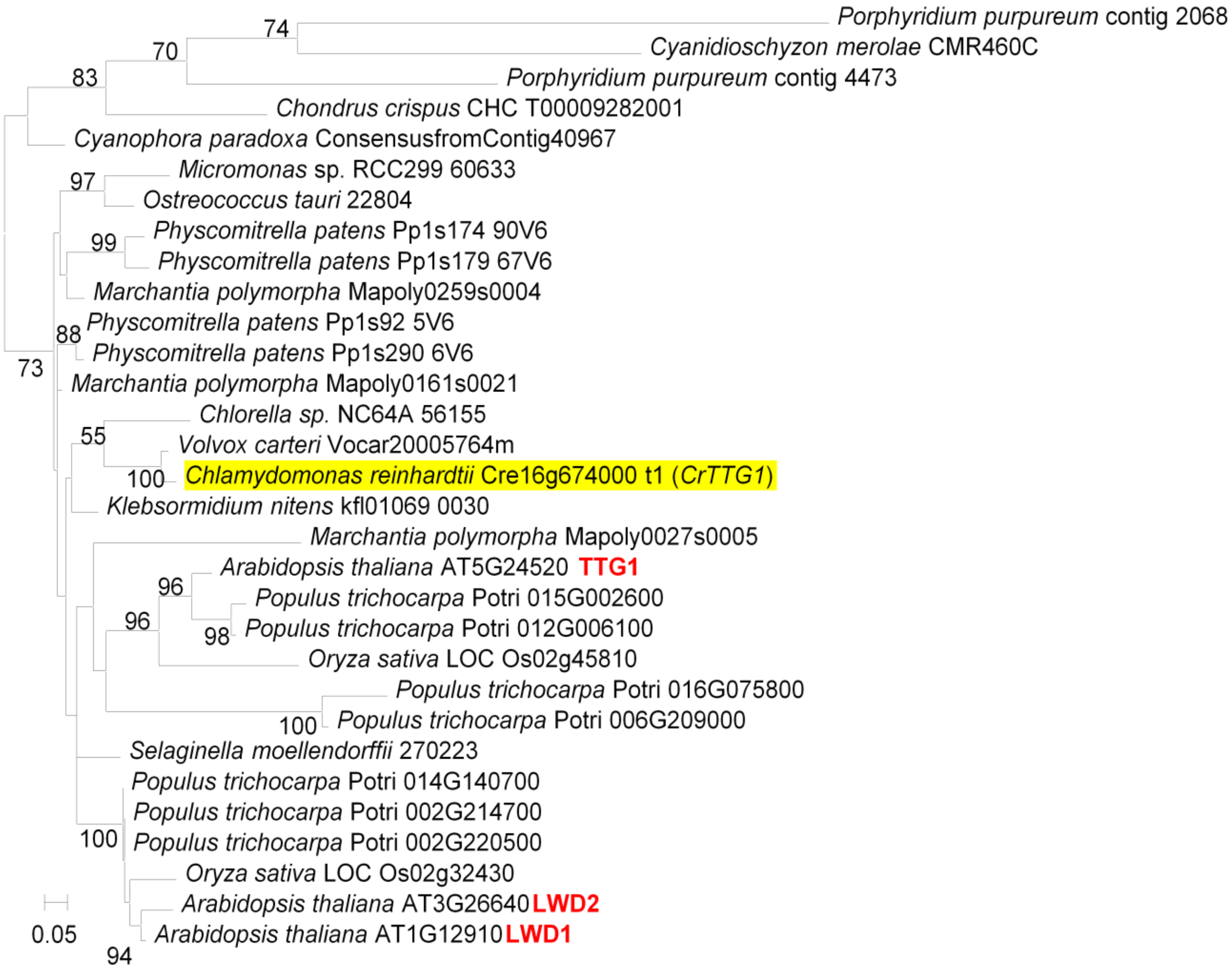

Porphyridium purpureum contig 2068
Cyanidioschyzon merolae CMR460C
Porphyridium purpureum contig 4473
Chondrus crispus CHC T00009282001
Cyanophora paradoxa ConsensusfromContig40967
Micromonas sp. RCC299 60633
Ostreococcus tauri 22804
Physcomitrella patens Pp1s174 90V6
Physcomitrella patens Pp1s179 67V6
Marchantia polymorpha Mapoly0259s0004
Physcomitrella patens Pp1s92 5V6
Physcomitrella patens Pp1s290 6V6
Marchantia polymorpha Mapoly0161s0021
Chlorella sp. NC64A 56155
Volvox carteri Vocar20005764m
Chlamydomonas reinhardtii Cre16g674000 t1 (CrTTG1)
Klebsormidium nitens kfl01069 0030
Marchantia polymorpha Mapoly0027s0005
Arabidopsis thaliana AT5G24520 TTG1
Populus trichocarpa Potri 015G002600
Populus trichocarpa Potri 012G006100
Oryza sativa LOC Os02g45810
Populus trichocarpa Potri 016G075800
Populus trichocarpa Potri 006G209000
Selaginella moellendorffii 270223
Populus trichocarpa Potri 014G140700
Populus trichocarpa Potri 002G214700
Populus trichocarpa Potri 002G220500
Oryza sativa LOC Os02g32430
Arabidopsis thaliana AT3G26640 LWD2
Arabidopsis thaliana AT1G12910 LWD1
74
70
83
97
99
88
73
55
100
96
96
98
100
100
94
0.05
