# Supplementary material for: Lipid remodeling regulator 1 (LRL1) is differently involved in the phosphorus‐depletion response from PSR1 in Chlamydomonas reinhardtii
Source: Plant J. 2019 Aug 23;100(3):610–26. doi: 10.1111/tpj.14473 (PMC6899820; doi:10.1111/tpj.14473)
Supplement: Supplementary file 2 — Table S1. List of RNA‐seq samples analyzed in this study. Table S2. Primers used in this study. [file TPJ-100-610-s002.pdf]

Table S1. List of RNA-seq samples analyzed in this study. RNA-seq method was described in detail in the experimental procedure section.

Sample[†]	Strain	Medium	Culture Period	Read length	Read number	Accession number[¶]
TAP4d_control	IAM-C9	TAP	4 days	Single 69 bp	12,543,990	DRX116072
TAP4d_*lrl1-1*	*lrl1-1*	TAP	4 days	Single 69 bp	9,348,971	DRX116073
TAP-P4d_control	IAM-C9	TAP -P	4 days	Single 69 bp	11,441,876	DRX116074
TAP-P4d_*lrl1-1*	*lrl1-1*	TAP -P	4 days	Single 69 bp	12,482,294	DRX116075
TAP-P2d_control	IAM-C9	TAP -P	2 days	Single 69 bp	11,956,123	DRX116076
TAP-P2d_*lrl1-1*	*lrl1-1*	TAP -P	2 days	Single 69 bp	9,423,354	DRX116077
TAP-P8d_CC-124	CC-124	TAP -P	8 days	Single 69 bp	20,519,974	DRX116078
TAP-P8d_CW15	CW15	TAP -P	8 days	Single 69 bp	16,656,143	DRX116079
TAP-P8d_CC-4334	CC4334	TAP -P	8 days	Single 69 bp	14,086,320	DRX116080
TAP-P13d_CC-124	CC-124	TAP -P	13 days	Single 69 bp	15,992,888	DRX116081
TAP-P13d_CW15	CW15	TAP -P	13 days	Single 69 bp	13,252,129	DRX116082
TAP-P13d_CC-4334	CC4334	TAP -P	13 days	Single 69 bp	14,568,809	DRX116083
TAP8d_CC-124	CC-124	TAP	8 days	Single 76 bp	58,148,100	DRX116084
TAP8d_CW15	CW15	TAP	8 days	Single 76 bp	55,054,153	DRX116085
TAP8d_CC-4334	CC4334	TAP	8 days	Single 76 bp	57,037,937	DRX116086
TAP13d_CC-124	CC-124	TAP	13 days	Single 76 bp	56,236,710	DRX116087
TAP13d_CW15	CW15	TAP	13 days	Single 76 bp	55,980,329	DRX116088
TAP13d_CC-4334	CC4334	TAP	13 days	Single 76 bp	53,125,076	DRX116089
TAP5d_CC-125	CC-125	TAP	5 days	Single 36 bp	18,895,977	DRX116090
TAP-P5d_CC-125	CC-125	TAP -P	5 days	Single 36 bp	26,813,509	DRX116091

[†]The sample naming is based on the culture condition (mixotrophic P-replete (TAP) or P-depleted (TAP–P) condition); period of culture; and strain.

[¶]The accession number refers to raw RNA-seq data deposited to The DNA Data Bank of Japan (DDBJ).

Table S2. Primers used in this study.

Primer name	Sequence 5' to 3'
ScPCR_Cre03.g197100_F1	TTATGACGGGGAAGGAAGCA
ScPCR_Cre03.g197100_F2	TAACATAAAGGGCGGCTGGT
ScPCR_Cre03.g197100_F3	AGTCTCCCAAGGTCGCTGGT
ScPCR_Cre03.g197100_F4	GACTTGCTCCCCTCATCACC
ScPCR_Cre03.g197100_F5	GGCTTGAGGCATGACGTAGA
ScPCR_Cre03.g197100_R1	TACTTTCGGGCAAAGCAAGG
ScPCR_Cre03.g197100_R2	GCCATTTCACACGCATTCTC
ScPCR_Cre03.g197100_R3	CAAACTGCATGTCCACAAACC
ScPCR_Cre03.g197100_R4	TGCAGGCGGATCATGTAGTT
ScPCR_Cre03.g197100_R5	GCGACTATGAGCGATTGGTG
Supp. Fig. 2-F1	AGCATACCCCGGGAAGGTGT
Supp. Fig. 2-R1	GCGACTATGAGCGATTGGTG
Supp. Fig. 2-F2	CCCGTCACCTTAACGCATC
Supp. Fig. 2-R2	AGCGACTCCTCCTCCTCAGT
Supp. Fig. 2-F3	ACAGAGGTGAAGCTAGGGCA
Supp. Fig. 2-R3	GTGCATGTCTTGCTGTTGCT
RB0-2	GTCGACTTGGAGGATCTGGACGA
RB-2	TACCGGCTGTTGGACGAGTTCTTCTG
CIB-1	GACGTTACAGCACACCCTTG
F-CDS	ATGTCCGGCGACAGCAGCGC
qPCR LRR1-F	TAACATAAAGGGCGGCTGGT
qPCR LRR1-R	GTTAAGGTGACGGGCGATG
qPCR DGTT1-F	CGCTGGGCTTCTCCAAA
qPCR DGTT1-R	ATGCCGTGCGATAGGATGT
qPCR MLDP-F	GGATGCCTGGACCAAGTTC
qPCR MLDP-R	GAGTGCACCAGGAGGTCGT
qPCR SQD2-1-F	CCTCAACTCTTCGCTGTCCAA
qPCR SQD2-1-R	TCACGCGGTTCGTATGC
qPCR SQD2-2-F	TCCACACCATCGTCAATCCA
qPCR SQD2-2-R	CGCACGGAAGATTGATCGTT
qPCR PTB4-F	CCAACCTGGCAATCTACATG
qPCR PTB4-R	GCCTTGTTCGAGTCCCAGT
qPCR PTB9-F	GCTCGCTCAACGCCTCTAAC
qPCR PTB9-R	GGTCGATGTAACCAATGCTCCT
qPCR CBLP-F	TGCTGTCCGTGGCTTTCTC
qPCR CBLP-R	GGCTCGCCAATGGTGTACTT

- Primers used for iVEC or infusion system

Primer name	Sequence 5' to 3'
ivec_pBI121_XhoI_fwd	TTCCCTCGAGATCAAAGGCCATGGAGTCAAAGA
ivec_pBI121_XhoI_rev	GTTGCTCGAGTGTCAGTTCCAAACGTAAAACGG
pBI121_left_ivec	GGCTACAGCCTCGGGAATTGCTACC
ivec_35S_rev	AAGGGACTGACCACCCGGGGATCCT
GUS_F	ATGTTACGTCCTGTAGAAACCCCAA
pBI121_right_ivec	GTGACTCCCTTAATTCTCCGCTCAT
ivec_p35S-TF-LRL1-F	AGGATCCCCGGGTGGTCAGTCCCTTATGTCCGGCGACAGCAGCGCCGG
ivec_p35S-TF-LRL1-R	GGTAGCAATTCCCGAGGCTGTAGCCTCACAGGCCGCCCGCCAGGCT
ivec_p35S-TF-PSR1-F	AGGATCCCCGGGTGGTCAGTCCCTTATGGACAAAGCTGAACGCGCTGC
ivec_p35S-TF-PSR1-R	GGTAGCAATTCCCGAGGCTGTAGCCCTATGGCTCCACTCGCTGCCGCTT
ivec_pSQD2-1-GUS-F	ATGAGCGGAGAATTAAGGGAGTCACGACGCTGGGACGTATTTCGCTGG
ivec_pSQD2-1-GUS-R	TTGGGGTTTCTACAGGACGTAACATTGTCGCTCTTGGCCCTCTAAAAGTAGC
ivec_pSQD2-2-GUS-F	ATGAGCGGAGAATTAAGGGAGTCACGCGTTGGGACGAGCCACGACA
ivec_pSQD2-2-GUS-R	TTGGGGTTTCTACAGGACGTAACATTTTGAAGTGTAGGTCCGCACGGAAGA
ivec_pLRL1-GUS-F	ATGAGCGGAGAATTAAGGGAGTCACGTCGACGCAACGCTACGCAAGTG
ivec_pLRL1-GUS-R	TTGGGGTTTCTACAGGACGTAACATAGTCGCGGGCTGCGCGCAAAGA
ivec_pPHT1-GUS-F	ATGAGCGGAGAATTAAGGGAGTCACCTCTGCTCACTTTGCGGTGCTTCTC
ivec_pPHT1-GUS-R	TTGGGGTTTCTACAGGACGTAACATCGTCGTCGCAGACGGACCCA
SUR1_left_ivec	ATGAGCGGAGAATTAAGGGAGTCACACATAGGCTCCAAATGGATGACAA
ivec_AtSUR1_pro_GUS_R	TTGGGGTTTCTACAGGACGTAACATCTTCTCTCTGTGCTTTGAGTTCTT

- Primers used for Gateway system

Primer name	Sequence 5' to 3'
AtMYB28_F	AAAAAGCAGGCTACAATGTCAAGAAAGCCATGTTGCGTC
AtMYB28_R	AGAAAGCTGGGTCATATGAAATGCTTTTCAAGCGAG
attB1-F	GGGGACAAGTTTGTACAAAAAAGCAGGCT
attB2-R	GGGGACCACTTTGTACAAGAAAGCTGGGT
